# *Vibrio vulnificus* induces the death of a major bacterial species in the mouse gut via cyclo-Phe-Pro

**DOI:** 10.1186/s40168-021-01095-w

**Published:** 2021-07-20

**Authors:** Jeong-A Kim, Bo-Ram Jang, Yu-Ra Kim, You-Chul Jung, Kun-Soo Kim, Kyu-Ho Lee

**Affiliations:** Department of Life Science, Sogang University, 35 Baekbeom-Ro, Mapo-Gu, Seoul, South Korea

**Keywords:** *Vibrio vulnificus*, Cyclo-Phe-Pro, Gut microbiota, *Bacteroides vulgatus*, Membrane disruption, ObgE

## Abstract

**Background:**

A foodborne pathogen, *Vibrio vulnificus*, encounters normal microflora inhabiting the gut environments prior to causing fatal septicemia or gastroenteritis and should overcome the barriers derived from the gut commensals for successful infection. Its interactions with gut commensals during the infection process, however, have not yet been understood. In the present study, the effect of *V. vulnificus* on the community structures of gut microbiota in mice was examined.

**Results:**

Analyses of microbiota in the fecal samples of mice that died due to *V. vulnificus* infection revealed the decreased abundance of bacteria belonged to Bacteroidetes, notably, the species *Bacteroides vulgatus*. In vitro coculturing of the two bacterial species resulted in the decreased survival of *B. vulgatus*. The antagonistic effect of *V. vulnificus* against *B. vulgatus* was found to be mediated by cyclo-Phe-Pro (cFP), one of the major compounds secreted by *V. vulnificus*. cFP-treated *B. vulgatus* showed collapsed cellular morphology with an undulated cell surface, enlarged periplasmic space, and lysed membranes, suggesting the occurrence of membrane disruption. The degree of membrane disruption caused by cFP was dependent upon the cellular levels of ObgE in *B. vulgatus*. Recombinant ObgE exhibited a high affinity to cFP at a 1:1 ratio. When mice were orally injected with cFP, their feces contained significantly reduced *B. vulgatus* levels, and their susceptibility to *V. vulnificus* infection was considerably increased.

**Conclusions:**

This study demonstrates that *V. vulnificus*-derived cFP modulates the abundance of the predominant species among gut commensals, which made *V. vulnificus* increase its pathogenicity in the hosts.

**Video abstract**

**Supplementary Information:**

The online version contains supplementary material available at 10.1186/s40168-021-01095-w.

## Introduction

During the infection processes of foodborne pathogens, they encounter numerous stresses through the gastrointestinal (GI) tract of the host. While surviving the fluctuating conditions of pH, oxygen, osmolarity, and nutrients [1, 2], they should also overcome the barriers of host epithelial structures and the antimicrobials produced by the host’s immune system [3, 4]. Therefore, successful infection requires the pathogens to perform cellular motility motivated by chemotactic sensing, penetration of the intestinal mucus layers, adherence to the epithelial surfaces, and colonization with production and/or secretion of virulence factors at the infection sites [5, 6]. In the case of *Vibrio vulnificus*, an opportunistic foodborne pathogen responsible for deaths worldwide due to seafood-associated septicemia [7], there have been numerous reports regarding its diverse virulence factors. In addition to well-known lipopolysaccharide and capsular polysaccharide, the membrane-bound and secreted proteins, including the immunogenic lipoprotein A (IlpA), hemolysin/cytolysin (VvhA), metalloprotease M (VvpM), phospholipase A2 (PlpA), and multifunctional-autoprocessing repeats-in-toxin (MARTX) show diverse activities to cause immunogenicity, cytotoxicity, and cell death via necrosis or apoptosis [8, 9]. Production of these virulence factors is strictly regulated by sensing the extracellular signals derived from hosts or bacteria to express them in appropriate conditions. Iron ions, an autoinducer (AI-2), and a cyclic dipeptide (cyclo-Phe-Pro [cFP]) have been listed as the main signal molecules involved in these regulations in *V. vulnificus*.

Most studies of *V. vulnificus* pathogenicity have focused on the bacterial effects on model animals at the levels of cells, tissues, or organs. Mammalian intestinal tracts are inhabited by an enormous amount of normal microflora made up of several hundred to a thousand species of bacteria, which primarily belong to the phyla Bacteroidetes, Firmicutes, Actinobacteria, and Proteobacteria [10, 11]. Interactions between pathogens and gut commensals have been reported in diverse pathogens in model systems. The antagonistic effects of gut commensals on entering pathogens are achieved via the restriction of resources available to pathogens, the competitive exclusion of pathogens from mucosal colonization, the inhibitory effects of antimicrobial compounds or appendages of the commensals, and the stimulation of host defense systems [12–16]. Therefore, the host animals, of which gut microbiomes experienced a change in their composition or abundance, showed altered susceptibility to foodborne pathogens. Increased pathogenicity in these host animals has been experimentally proven in infections by *Salmonella enterica* Typhimurium, *Listeria monocytogenes*, *V. cholerae,* and a pathogenic *Escherichia coli* [17–20]. Furthermore, some pathogens have shown the ability to affect gut microbiota by direct killing with the type VI secretion systems or bacteriocins [21, 22].

The results of these previous studies lead us to speculate the existence of a similar situation where foodborne *V. vulnificus* encounters the normal microflora in the guts. Therefore, this pathogen should interact with them to overcome the potential defense mechanisms directly or indirectly derived from the gut microbiota, while interacting with the hosts via timely produced virulence factors. In the present study, we observed dramatic changes in the gut microbiomes of mice upon infection by *V. vulnificus*, in which the levels of one of the representative gut commensals, *Bacteroides vulgatus*, were considerably decreased. The molecular characteristics in the decreased survival of *B. vulgatus* by *V. vulnificus* were delineated by demonstrating the specific interaction of a signal molecule, cFP of *V. vulnificus* with a putative GTPase protein in *B. vulgatus*.

## Results

### Reduced levels of *B. vulgatus* in the fecal microbiota of the *V. vulnificus*-infected mice

**Fig. 1:**
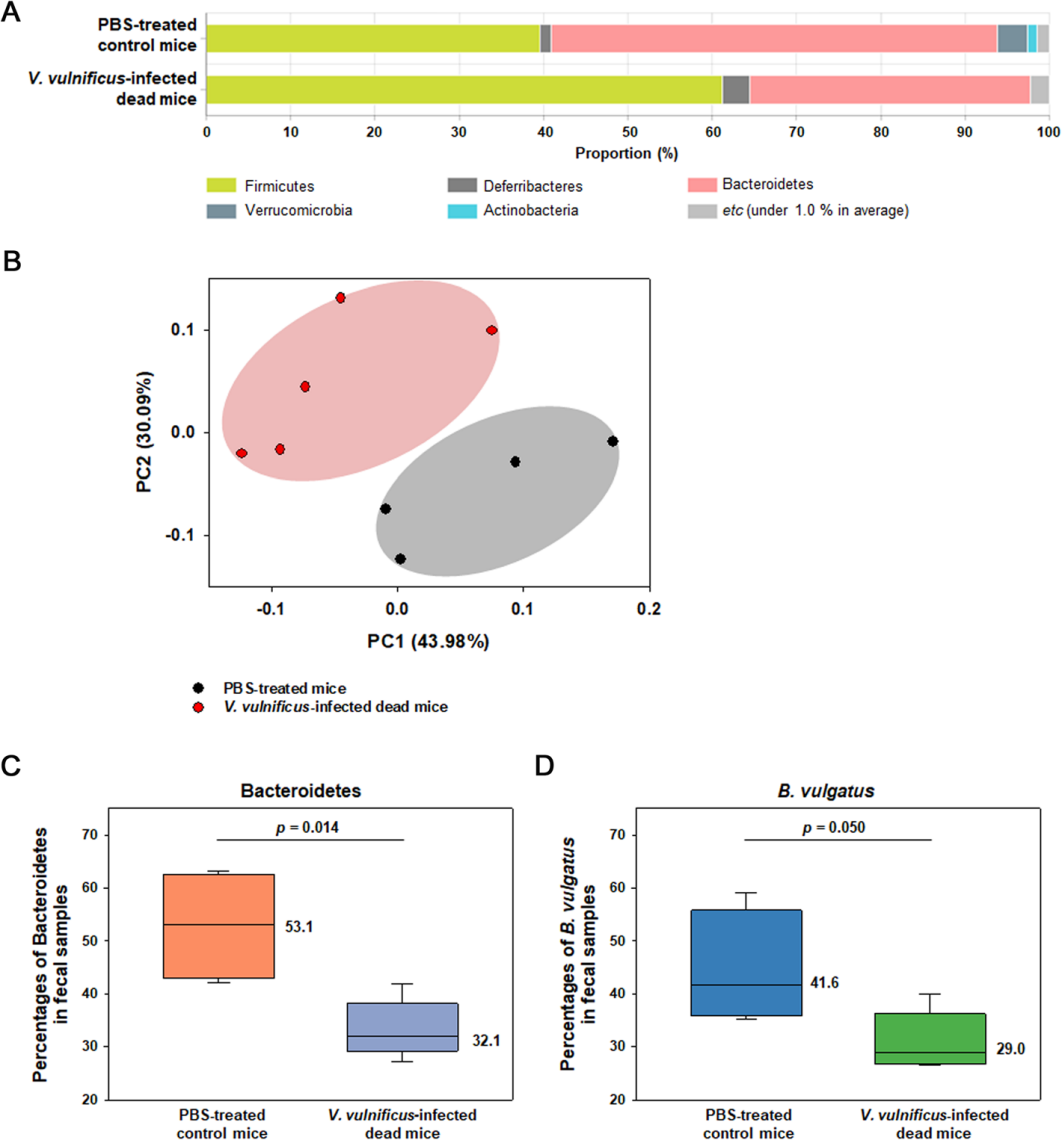
Microbiome analyses of fecal samples collected from *V. vulnificus*-infected mice. **A** Abundance of the bacterial phyla in fecal samples of *V. vulnificus*-infected mice. After the oral infection of mice with *V. vulnificus*, their fecal samples were collected from the cages individually containing dead mice (*n* = 5). Mice orally injected with *V. vulnificus*-free PBS were used as a negative control (*n* = 4). Each fecal sample was subjected to 16S rDNA sequencing to analyze the microbial community as described in “Methods.” The bacterial phyla are differently colored as indicated below the bar chart. All phyla comprising less than 1.0 % of the total abundance (whether classified or not) were combined into the “etc” category. **B** Principal coordinate analysis (PCoA) clustering of the bacterial communities in the mouse fecal samples. Community structures of microbiome derived from sequencing analysis of 16S rDNAs in the fecal samples of *V. vulnificus*-infected dead mice and PBS-treated control mice were subjected to PCoA based on Jensen-Shannon distance method. A resultant plot was generated across the samples, in which the black and red circles represent the species composition of PBS-treated mice and *V. vulnificus*-infected dead mice, respectively (PERMANOVA, *P* = 0.024). **C** and **D** Comparison of relative abundance of Bacteroidetes and *Bacteroides vulgatus* in the mouse fecal samples. The percentages of 16S rDNA copies belonged to the phylum Bacteroidetes (**C**) and the species of *B. vulgatus* (**D**) in the fecal samples of *V. vulnificus*-infected dead mice were compared with those of PBS-treated control mice. Their distribution was plotted with each median value, and statistical significance between two groups was determined using the Mann-Whitney *U* test

Fecal samples were collected from mice, which had been subjected to gavage with *V. vulnificus* and died within 20 h post-infection. Two independent sets of infection experiments were performed, and a total of 5 fecal samples were retrieved (three and two animals died in experiments 1 and 2, respectively). As a negative control, fecal samples from healthy mice treated with *V. vulnificus*-free PBS were also collected at each experimental set (two animals in each set). Analysis of 16S rDNA sequences amplified from the prokaryotic cells of the domain Bacteria in fecal samples showed that Bacteroidetes and Firmicutes were the major phyla occupying approximately 53% and 40% of total fecal microbiomes in the control mice, respectively (Fig. 1A). However, altered compositions of the two phyla were found in the *V. vulnificus*-fed mice: Although these two phyla were still the major phyla, Bacteroidetes and Firmicutes occupied approximately 33% and 61% of total fecal microbiomes, respectively (Fig. 1A). A principal coordinate analysis (PCoA) showed that the microbial compositions in most fecal samples of the *V. vulnificus*-fed mice were distinct from those of the control mice (permutational multivariate analysis of variance [PERMANOVA], *P* = 0.024) (Fig. 1B).

When the percentages of Bacteroidetes in the fecal samples were analyzed, their median values were 53.1% and 32.1% in the control mice and *V. vulnificus*-fed mice, respectively (Fig. 1C), and the difference between the two groups was significant (Mann-Whitney *U* test, *P* = 0.014). Among the bacteria belonging to Bacteroidetes, *B. vulgatus*, which has been recently reclassified as *Phocaeicola vulgatus* [23], was shown to be the most abundant species in mouse feces in both sets of experiments (Additional file: Table S1). The predominant abundance of *B. vulgatus* was consistent with the data previously reported [20]. Median values of the percentages of *B. vulgatus* in control mice and *V. vulnificus*-fed mice were 41.6% and 29.0%, respectively (Fig. 1D). Reduced levels of *B. vulgatus* were observed in all of the *V. vulnificus*-fed mice in each set of experiments (Mann-Whitney *U* test, *P* = 0.050): 52.5% ± 9.5% in the control vs. 33.2% ± 6.5% in the infected mice in experiment 1; and 36.4% ± 1.5% in the control vs. 27.8% ± 1.8% in the infected mice in experiment 2 (Additional file: Table S1). In addition to *B. vulgatus*, another bacterial species belonged to Bacteroidetes such as *Parabacteroides goldsteinii* also decreased in the *V. vulnificus*-fed mice (Additional file: Table S1). In contrast, some *Lactobacillus* species, which were the main bacteria belonging to Firmicutes in the mouse guts, did not show a decrease in their levels in *V. vulnificus*-fed fecal samples, but these increased in some animals.

### Decreased viability of *B. vulgatus* in the presence of *V. vulnificus*

**Fig. 2:**
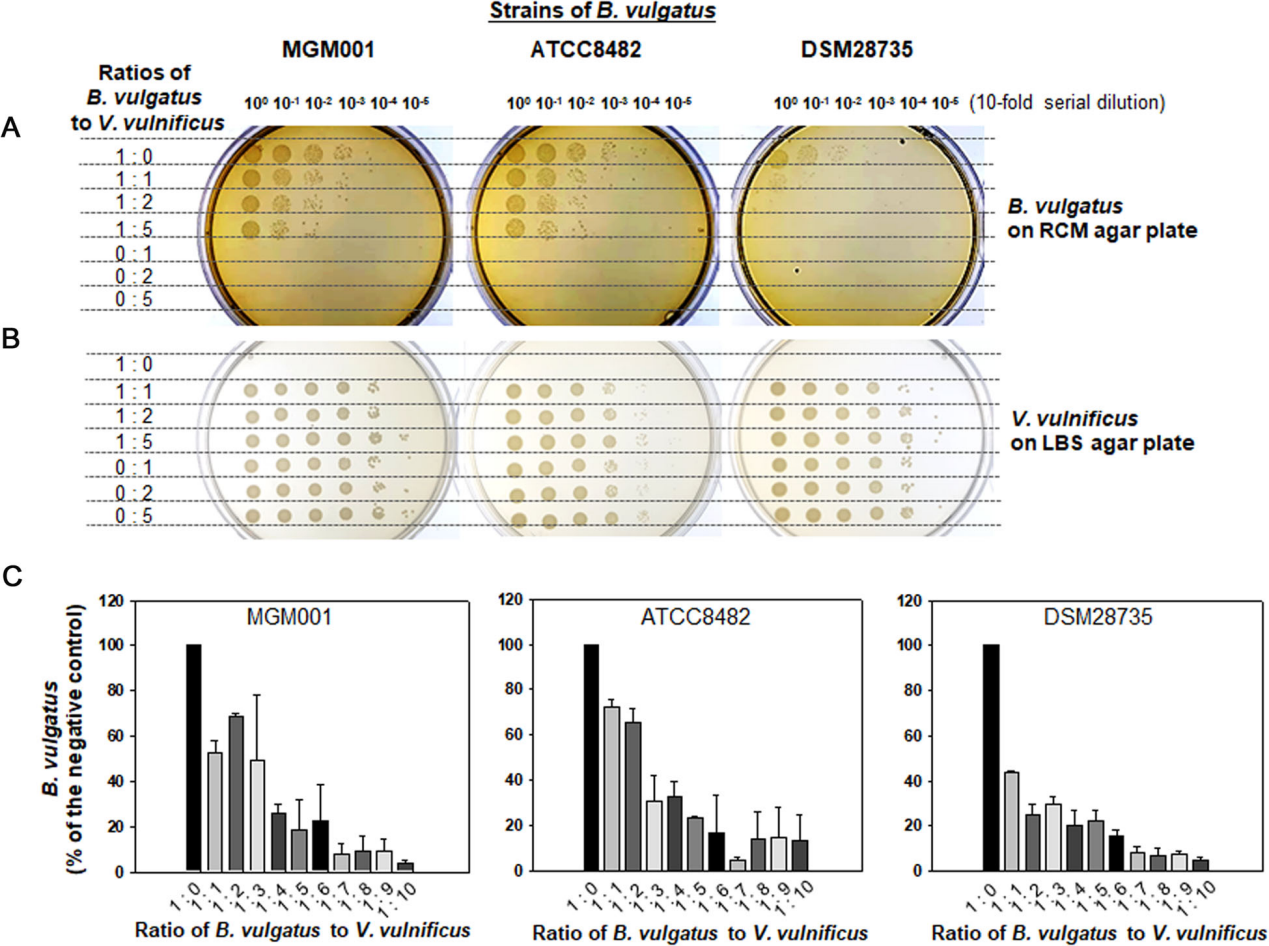
Survival of *B. vulgatus* in the presence of *V. vulnificus*. **A** and **B** Spot inoculation of serially diluted cocultures of *B. vulgatus* and *V. vulnificus*. Three strains of *B. vulgatus*, MGM001, ATCC8482, and DSM28735, were coincubated with *V. vulnificus* in the M9-minimal medium. The ratios of *B. vulgatus* to *V. vulnificus* in cocultures were 1:1, 1:2, and 1:5. Each mixture was incubated in a 37 °C anaerobic chamber for 6 h. To compare the survival of each species, aliquots of serially-diluted cultures were spotted on RCM agar plate for *B. vulgatus* (**A**) and LBS agar plate for *V. vulnificus* (**B**). For validation of the selective growth of two species of bacteria on each agar plate, the monocultures containing either *B. vulgatus* (designated as 1:0) or *V. vulnificus* (designated as 0:1, 0:2, and 0:5) were included. **C** Estimation of viable *B. vulgatus* cells in the presence of various concentrations of *V. vulnificus*. Various combinatory ratios of *B. vulgatus* to *V. vulnificus*, ranging from 1:1 to 1:10, were prepared in the M9-minimal medium and incubated in a 37 °C anaerobic chamber. After 6 h, the CFUs of *B. vulgatus* were enumerated by spreading aliquots of serially-diluted cultures on the RCM agar plates. The percentage of *B. vulgatus* CFUs in each mixture was obtained by dividing them by the number of *B. vulgatus* CFUs in the control (*B. vulgatus* only; designated by 1:0 on the x-axis)

To examine whether the decrease in *B. vulgatus* levels in the *V. vulnificus*-fed mice was caused by an interaction between these two species of bacteria, a strain of *B. vulgatus* (MGM001; Additional file: Table S2) was isolated as described in “Methods.” In addition to MGM001, two strains of *B. vulgatus* were purchased from the American Type Culture Collection (ATCC) and Deutsche Sammlung von Mikroorganismen und Zellkulturen (DSMZ). Three strains of *B. vulgatus* were anaerobically incubated in the M9-minimal medium with *V. vulnificus*. After 6 h of coincubation of *B. vulgatus* with *V. vulnificus* at ratios of 1:1, 1:2, and 1:5, serially diluted cocultures were spot-inoculated on RCM agar plates and incubated in an anaerobic chamber for the growth of *B. vulgatus* (Fig. 2A). The same aliquots were spot-inoculated on LBS agar plates for *V. vulnificus* growth (Fig. 2B). For comparisons of the viable cells of each species in the monocultures, these were also prepared to contain either *B. vulgatus* only (designated as 1:0) or *V. vulnificus* only (designated as 0:1, 0:2, and 0:5). The viability of all *B. vulgatus* strains tested in this study decreased in the presence of *V. vulnificus*, of which reduction appeared to be dependent on the abundance of *V. vulnificus* in cocultures. On the other hand, the viability of *V. vulnificus* was not affected by *B. vulgatus*.

To examine the dose-dependent reduction in *B. vulgatus* viability, the same amount of *B. vulgatus* was incubated under the conditions of various amounts of *V. vulnificus*, at ratios of *B. vulgatus* to *V. vulnificus* ranging from 1:1 to 1:10 (Fig. 2C). After 6 h, viable members of *B. vulgatus* were enumerated by spreading aliquots of serially-diluted cultures on the RCM agar plates. The results showed that the effect of *V. vulnificus* on *B. vulgatus* viability was antagonistic in a dose-dependent manner. The responses of the three strains of *B. vulgatus* to *V. vulnificus* were evaluated by comparing the ratios of *B. vulgatus* to *V. vulnificus*, which resulted in 50% reduction of viable cells of *B. vulgatus*: Calculated ratios of MGM001, ATCC8482, and DSM28735 to *V. vulnificus* were 1:2.0, 1:2.1, and 1:1.0, respectively. The strain MGM001, which has been isolated in this study, was used for the subsequent investigations.

### Identification of an antagonistic compound in the *V. vulnificus*-spent medium

**Fig. 3:**
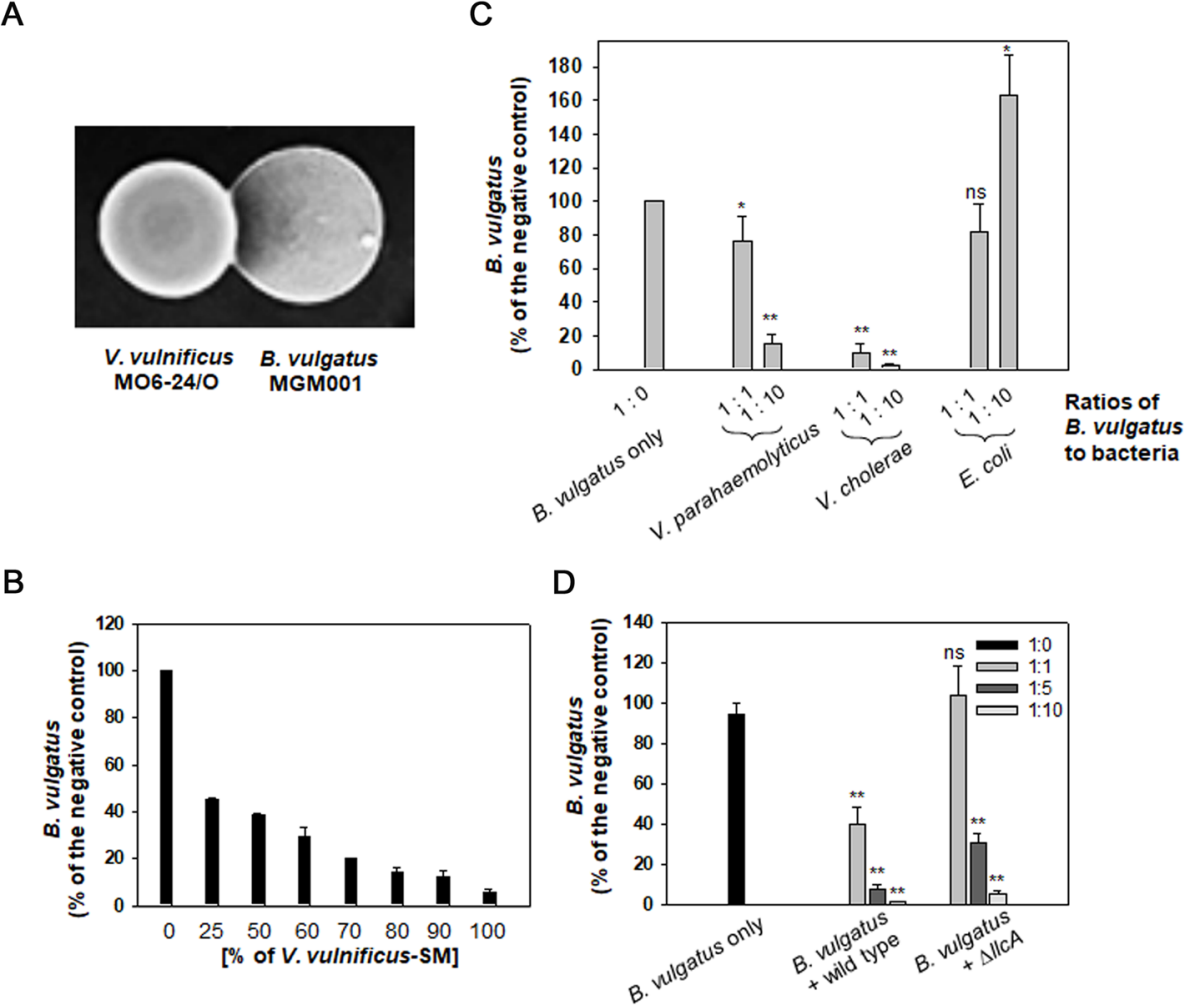
Effects of various components in the supernatants of *V. vulnificus*-cultures on the survival of *B. vulgatus*. **A** Interaction between colonies of *V. vulnificus* and *B. vulgatus*. The resuspensions of the freshly grown cells of *V. vulnificus* and *B. vulgatus* were spotted in vicinity on a RCMS agar plate. After 24 h of anaerobic incubation at 37 °C, the growth pattern of each colony was observed. **B** Survival of *B. vulgatus* in the presence of cell-free supernatants of *V. vulnificus*. The cell-free supernatants (spent medium, SM) of *V. vulnificus* cultured in LBS for 18 h were prepared as described in the “Methods” section. Serially diluted SM with the fresh LBS broth was added to *B. vulgatus*. After 6 h anaerobic incubation at 37 °C, *B. vulgatus* CFUs were enumerated as described in Fig. 2C. **C** Survival of *B. vulgatus* in the presence of *Vibrio* spp. and *E. coli*. *B. vulgatus* suspensions in M9-minimal medium were mixed with *V. parahaemolyticus, V. cholerae*, or *E. coli* at ratios of 1:1 and 1:10 (*B. vulgatus* to other bacteria), and incubated in a 37 °C anaerobic chamber for 6 h. After 6 h, the CFUs of *B. vulgatus* were enumerated as described in “Methods.” The *P*-values for the comparison with the control (*B. vulgatus* only) are indicated (Student’s *t*-test; ns, not significant; *0.001 < *P* < 0.01; **P < 0.001). **D** Survival of *B. vulgatus* in the presence of a mutant *V. vulnificus* producing less cFP. An *llcA*-defective *V. vulnificus*, which has been shown to produce significantly lowered amounts of cFP [24], was mixed with *B. vulgatus* at ratios of 1:1, 1:5, and 1:10 (*B. vulgatus*:*V. vulnificus*). *B. vulgatus* CFUs were enumerated as described in Fig. 2C

To examine how *V. vulnificus* affected the viability of *B. vulgatus*, both bacteria were spotted on a RCMS (salt-enriched RCM) agar plate, on which two bacterial colonies grew and were in contact with each other. Resultant interaction between the two colonies showed the presence of a growth inhibition zone of the *B. vulgatus* colony confronting the *V. vulnificus* colony (Fig. 3A). This suggested that the inhibition of *B. vulgatus* growth may have been mediated by a direct interaction with *V. vulnificus* cells and/or an indirect interaction via secreted compounds from *V. vulnificus*. To test these hypotheses, the involvement of the type VI secretion system (T6SS) of *V. vulnificus* in limiting the growth of *B. vulgatus* via direct contact, was examined. A mutant (Δ*icmF*) deficient in one of the T6SS components of *V. vulnificus*, i.e., IcmF (the intracellular multiplication factor F [25];), was constructed and mixed with *B. vulgatus* cells. However, it showed the same degrees of *B. vulgatus* survival shown by the wild-type *V. vulnificus* (Additional file: Figure S1A), which suggested that *B. vulgatus* death was not caused by direct cell-to-cell interaction via T6SS of *V. vulnificus*.

Next, the cell-free supernatant derived from the LBS broth grown by *V. vulnificus* to the stationary phase (spent medium, SM) was prepared and various volumes of the filter-sterilized SM were added to *B. vulgatus* (Fig. 3B). Compared to the control, 0% of SM (the fresh LBS medium only), the addition of SM resulted in a decrease in *B. vulgatus* viability in a dose-dependent manner. This suggested the possible role of *V. vulnificus*-originating and *V. vulnificus*-secreted substance(s) in the death of *B. vulgatus*. To screen the active components in SM, the major metabolites produced by *V. vulnificus* under anaerobic conditions, including pyruvate, formate, lactate, and acetate [26], were added to a suspension of *B. vulgatus* cells (Additional file: Figure S1B). None were shown to be effective in decreasing *B. vulgatus* survival to a concentration of at least 20 mM. In addition, the heated SM, which had been treated at 95 °C for 5 min to denature its proteineous substances, was mixed with *B. vulgatus* (Additional file: Figure S1C). However, the heat-treated SM showed a similar effect to that of the original SM.

To circumvent the difficulty in finding the active compound(s), *B. vulgatus* was challenged by various bacterial species and its survival was examined (Fig. 3C). All the *Vibrio* species tested in this study showed antagonistic effects on *B. vulgatus*. Among them, *V. cholerae* was the most effective in decreasing the survival of *B. vulgatus*, since the incubations containing bacterial cells composed of a 1:1 ratio showed similar levels to those shown by the incubations containing 10 times more *V. parahaemolyticus*. In contrast, *E. coli* strain S17-1 s*pir* did not exhibit this effect but instead slightly increased the *B. vulgatus* levels in the 1:10 incubation. Three *Vibrio* species tested in this study have been shown to produce a cyclic dipeptide, cFP, in their SM, and the concentration of cFP in the *V. vulnificus* SM has been estimated to be up to 0.9 mM [27]. In addition, the incubation of *B. vulgatus* with a mutant *V. vulnificus* (Δ*llcA*), of which production of cFP was estimated to be less than ~ 30% of the wild type [24], resulted in lowered effectiveness in decreasing *B. vulgatus* survival, compared to that with the same amount of wild-type *V. vulnificus* (Fig. 3D). These results suggested that the death of *B. vulgatus* might be mediated by cFP secreted to SM.

**Fig. 4:**
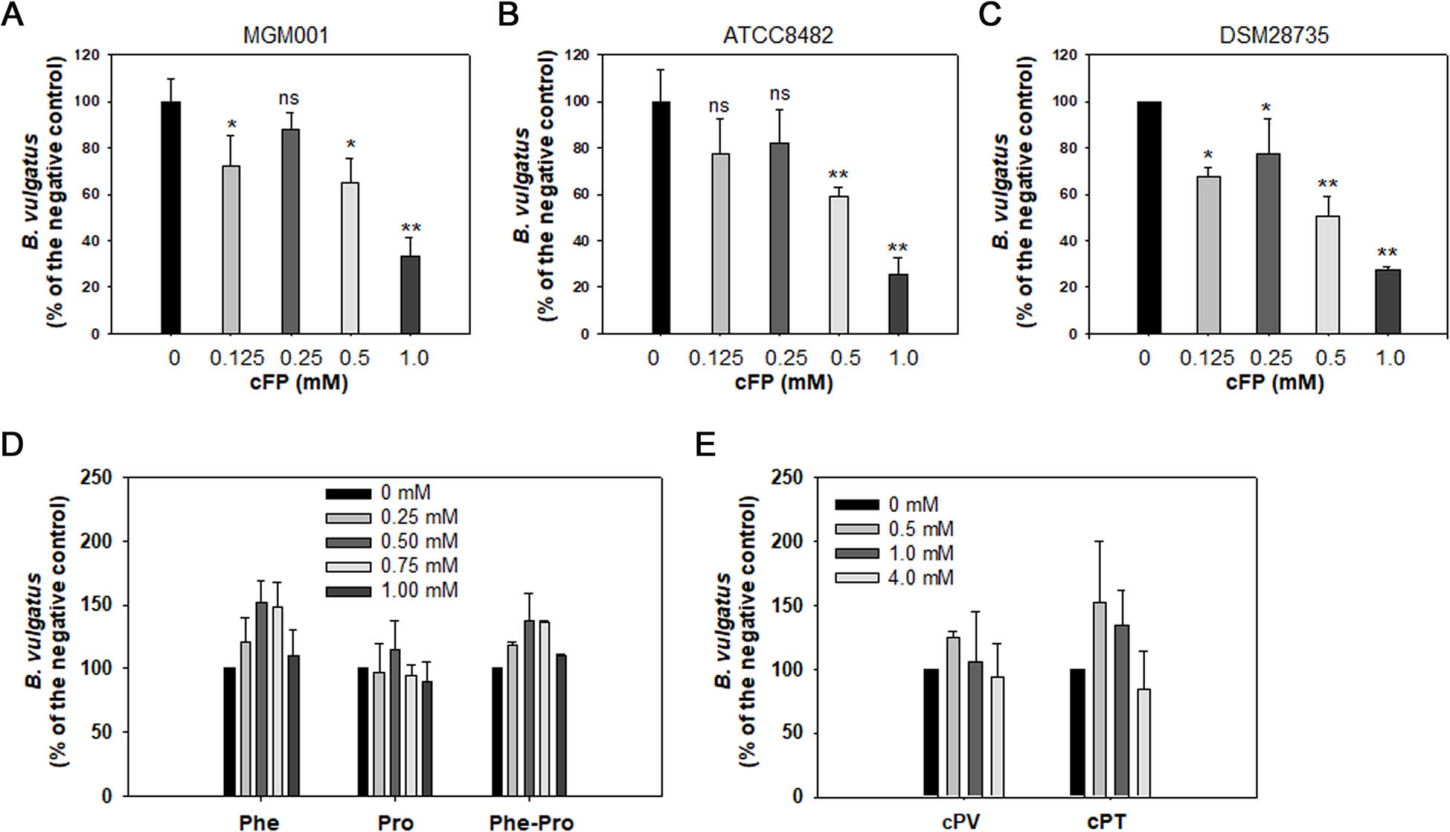
Effects of exogenous addition of cyclic dipeptides on the survival of *B. vulgatus*. **A**, **B**, and **C** Effect of cyclo-Phe-Pro (cFP) on the survival of the strains of *B. vulgatus*. Various concentrations of cFP ranging from 0 to 1.0 mM were added to the freshly grown cultures of three strains of *B. vulgatus* in M9 broth: MGM001 (**A**), ATCC8482 (**B**), and DSM28735 (**C**). At 6 h post-exposure to cFP, *B. vulgatus* CFUs were enumerated as described in Fig. 2C. The *P*-values for the comparison with the control (0 mM cFP) are indicated (Student’s *t*-test; ns, not significant; *0.001 < *P* < 0.01; **P < 0.001). **D** Effects of Phe (F), Pro (P), and a dipeptide of Phe-Pro (F-P) on the survival of *B. vulgatus*. Various concentrations of F, P, and F-P ranging from 0 to 1.0 mM were added to the freshly grown culture of *B. vulgatus* MGM001. At 6 h post-exposure, *B. vulgatus* CFUs were enumerated as described in Fig. 2C. **E** Effects of other cyclic dipeptides on the survival of *B. vulgatus*. Various concentrations of cyclo-Phe-Val (cPV) and cyclo-Pro-Thr (cPT) ranging from 0 to 4.0 mM were added to the freshly grown culture of *B. vulgatus* MGM001. At 6 h post-exposure, *B. vulgatus* CFUs were enumerated as described in Fig. 2C

To verify this, various concentrations of cFP, ranging from 0.125 to 1.0 mM, were added to the suspensions of three strains of *B. vulgatus* and incubated for 6 h (Fig. 4A–C). The survival of all strains was significantly affected by cFP in a concentration-dependent manner. However, when a single amino acid, F or P, and the linear dipeptide F-P were added to *B. vulgatus* cells at the same concentrations used for cFP, they did not show any effect on *B. vulgatus* survival (Fig. 4D). Furthermore, other cyclic dipeptides, such as cyclo(Phe-Val) (cPV) and cyclo(Pro-Thr) (cPT), did not show any antagonistic result up to a concentration of 4 mM (Fig. 4E). These results suggested that the *V. vulnificus*-originated cFP determined the specificity of the interaction between *B. vulgatus* and *V. vulnificus*.

### Induction of *B. vulgatus* cell death by cFP

**Fig. 5:**
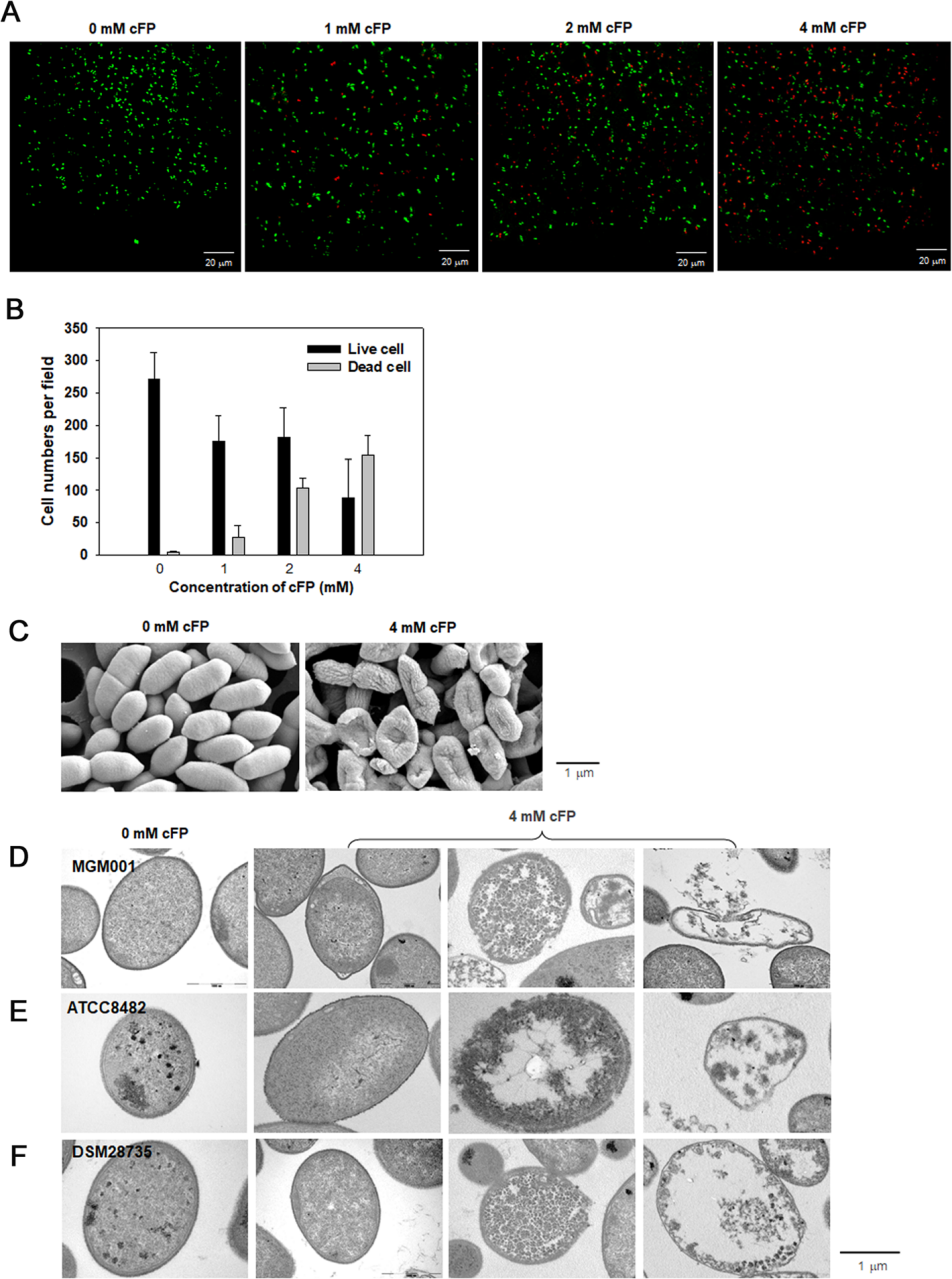
Cell death of *B. vulgatus* in the presence of cFP. **A** and **B** Live/Dead staining of *B. vulgatus* cells. Cells of *B. vulgatus* MGM001 were treated for 6 h with various concentrations of cFP ranging from 0 to 4.0 mM, and then stained with the Live/Dead cell double staining kit (**A**). Live and dead cells were enumerated by counting the green- and red-stained cells in at least eight fields, respectively, and their averages and standard deviations were plotted against the exposed concentrations of cFP (**B**). **C** SEM of *B. vulgatus* cells. *B. vulgatus* MGM001 was treated with 4.0 mM cFP for 6 h, then processed for SEM analysis as described in “Methods.” The length of a horizontal bar represents 1 μm. **D**, **E**, and **F** TEM of *B. vulgatus* cells. Four millimolar of cFP was added to the freshly grown cultures of three *B. vulgatus* strains; MGM001 (**B**), ATCC8482 (**C**), and DSM28735 (**D**). At 6 h post-exposure to cFP, *B. vulgatus* cells were processed for TEM analysis as described in “Methods.” For comparison, TEM images of the control cells (0 mM cFP) were presented in the left panels. The length of a horizontal bar represents 1 μm

To directly observe the effect of cFP on *B. vulgatus* cells, the strain MGM001 was treated for 6 h with various concentrations of cFP ranging from 0 to 4.0 mM, and then stained using a Live/Dead cell double staining kit (Sigma-Aldrich) (Fig. 5A). Through fluorescence microscopy, the number of red-stained dead cells were found to increase with cFP concentration, while the number of green-stained live cells gradually decreased (Fig. 5B).

The increased death of *B. vulgatus* observed via staining with fluorescence dyes was confirmed using electron microscopes. Scanning electron microscopy (SEM) of the MGM001 cells (Fig. 5C), which were treated with 4.0 mM cFP for 6 h, showed the collapsed cellular morphology with undulated cell surfaces. In addition, transmission electron microscopy (TEM) of three *B. vulgatus* strains presented dramatic alteration in cellular morphology upon treatment with cFP, with enlarged periplasmic space, lyzed membranes, and partially ruptured cells (Fig. 5D–F). It indicated the occurrence of membrane disruption in the cFP-treated cells.

### Effects of cFP on *Parabacteroides* and *Lactobacillus*

**Fig. 6:**
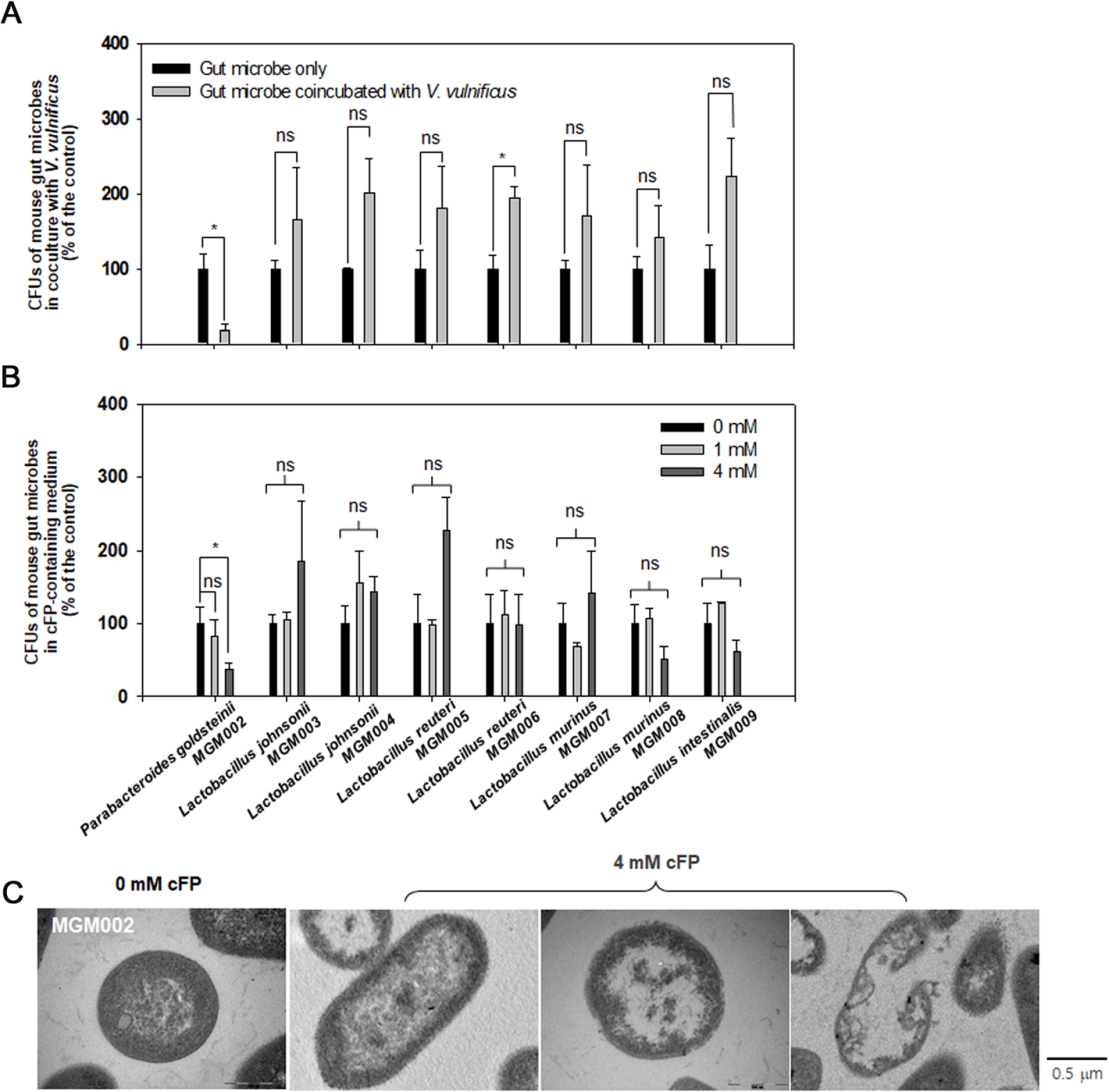
Responses of various MGM isolates to *V. vulnificus* and cFP. **A** and **B** Effects of *V. vulnificus* cells and cFP on the survival of MGM strains. Various bacterial strains (MGM isolates listed in the Additional file: Table S2) were incubated under the conditions with 5.0 × 10^9^ cells of *V. vulnificus* (**A**) or supplemented with cFP at concentrations of 1 and 4 mM (**B**). At 6 h of incubation, CFUs of the MGM strains were enumerated and presented with the percentage of each control (the same cultures but without treatment of *V. vulnificus* (**A**) or cFP (**B**)). The *P*-values for the comparison with the control (MGM only) are indicated (Student’s *t*-test; ns, not significant; *0.001 < *P* < 0.01). **C** TEM of *P. goldsteinii* cells. Four millimolar cFP was treated to the freshly grown culture of *P. goldsteinii* MGM002. At 6 h post-exposure to cFP, cells were processed for TEM analysis as described in the “Methods.” For comparison, a TEM image of no cFP-treated cells was presented in the left panel. The length of a horizontal bar represents 0.5 μm

From an effort to obtain pure cultures of microorganisms from mouse guts, another bacterial species, *P. goldsteinii* belonging to Bacteroidetes and several *Lactobacillus* species belonging to Firmicutes have been isolated (mouse gut microorganism (MGM) isolates; Additional file: Table S2). For the addition of *V. vulnificus* cells (2.0 × 10^8^ cells; Fig. 6A) or cFP (1 and 4 mM; Fig. 6B) to the resuspensions of the isolated gut commensals, only *P. goldsteinii* experienced decreased survival, while the survival of *Lactobacillus* species (i.e., *L. johnsonii*, *L. reuteri*, *L. murinus*, and *L. intestinalis*) was not decreased. Observation of cFP-treated *P. goldsteinii* cells under TEM showed apparent membrane disruption (Fig. 6C), as shown in the cFP-treated *B. vulgatus*.

### cFP-induced cell death of *B. vulgatus* via membrane disruption

**Fig. 7:**
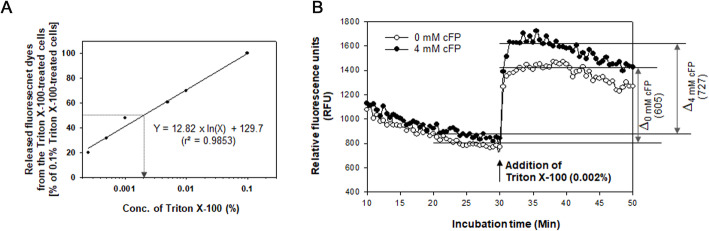
Effect of cFP exposure on the membrane permeability of *B. vulgatus*. **A** Estimation of the EC_50_ of Triton X-100. Various concentrations of Triton X-100, ranging from 0.0003% to 0.1%, were exposed to *B. vulgatus* MGM001 cells, which were incorporated with a membrane-associating fluorescent dye, DiSC3(5), as described in “Methods.” Released fluorescence from the N% Triton X-100-treated cells (termed Δ_Ν%_) was obtained by subtracting the values of basal RFU (before treatment of N% Triton X-100) from the maximal RFU (after treatment of N% Triton X-100). These values were normalized with the estimates of DiSC3(5) released from the cells treated with 0.1% Triton X-100 (Δ_0.1%_), at which almost all *B. vulgatus* cells were permeabilized. The ratios ([Δ_Ν%_/Δ_0.1%_] × 100) were plotted against the concentrations of Triton X-100. The arrow indicates the effective concentration (EC_50_) of Triton X-100 to release 50% of Δ_0.1%_. **B** Release of DiSC3(5) from cFP-treated *B. vulgatus*. A fluorescent dye, DiSC3(5), was used to examine the change in permeability of bacterial membrane. *B. vulgatus* cells, which had been exposed to 4 mM cFP for 6 h, were mixed with 0.4 μΜ DiSC3(5) for 1 h. After unincorporated dyes were washed out, RFUs from DiSC3(5) associated with cells were measured. At 30 min (as indicated with a black vertical arrow), cells were treated with 0.002% Triton X-100, at which approximately 50% of the *B. vulgatus* cells were permeabilized (as shown in **A**). The released fluorescence from the Triton X-100-treated cells was obtained by subtracting the values of basal RFUs (averaged RFUs for 10 min before treatment of 0.002% Triton X-100) from the maximal RFUs (averaged RFUs for 10 min after treatment of 0.002% Triton X-100). As a control, the cells untreated with cFP were followed by the same procedure and then Δ_0 mM cFP_ was compared with Δ_4 mM cFP_. Each line was derived from the average values from the total six experiments

To examine the changes in membrane integrity, a fluorescent dye, 3,3′-dipropylthiadicarbocyanine iodide (DiSC3(5)), was used to incorporate into bacterial membranes [28]. Then, the amount of DiSC3(5) released from bacterial cells was measured upon cell lysis by exposure to a membrane permeabilizing agent, such as Triton X-100. Total amounts of the membrane-associated DiSC3(5) were estimated by measuring the fluorescence released from the cells treated with 0.1% Triton X-100, for which almost all *B. vulgatus* cells were lysed. To determine the effective concentration of Triton X-100 causing the lysis of 50% *B. vulgatus* cells (EC_50_), various concentrations of Triton X-100 were exposed to DiSC3(5)-associated *B. vulgatus*. By plotting the percentages of dyes normalized with the estimate of released DiSC3(5) at 0.1% Triton X-100, as described in “Methods,” EC_50_ was calculated to be approximately 0.002% (Fig. 7A). The released fluorescence upon exposure to 0.002% Triton X-100 from *B. vulgatus* cells treated with 4 mM cFP (termed by Δ_4 mM cFP_) and the control *B. vulgatus* cells (termed by Δ_0 mM cFP_) was estimated (Fig. 7B). The values of Δ_0 mM cFP_ and Δ_4 mM cFP_ were estimated at 605 (± 64) and 727 (± 80) RFUs, which was equivalent to 29% (± 2.3%) and 43% (± 3.7%) of the total fluorescence initially incorporated into the cells, respectively. This difference was significant with a *P* value less than 0.008 (Student’s *t*-test). These results evidencing the increased susceptibility of cFP-treated cells to Triton X-100 suggested that cFP caused the membranes of *B. vulgatus* to be permeable, thus easily disruptive.

To differentiate whether this increased susceptibility to a membrane permeabilizing stress was specifically mediated by cFP or caused by any dipeptide composed of hydrophobic amino acids, the same experiments were performed using 4 mM of FP, cPT, and cPV (Additional file: Figure S2). *B. vulgatus* cells treated with FP, cPT, or cPV showed a slightly lowered basal RFU (averaged RFUs for 10 min before Triton X-100 treatment) compared to the control and much lower maximal RFU (averaged RFUs for 10 min after Triton X-100 treatment) than the cFP-treated cells. Upon the addition of 0.002% Triton X-100, *B. vulgatus* cells treated with these dipeptides produced Δ_4χmM dipeptide_ values (562, 572, and 561 RFUs in treatment with FP, cPT, and cPV, respectively) with similar ranges to that of the control cells (608 RFUs).

### Identification of a *B. vulgatus* factor involved in cFP-mediated membrane disruption

**Fig. 8:**
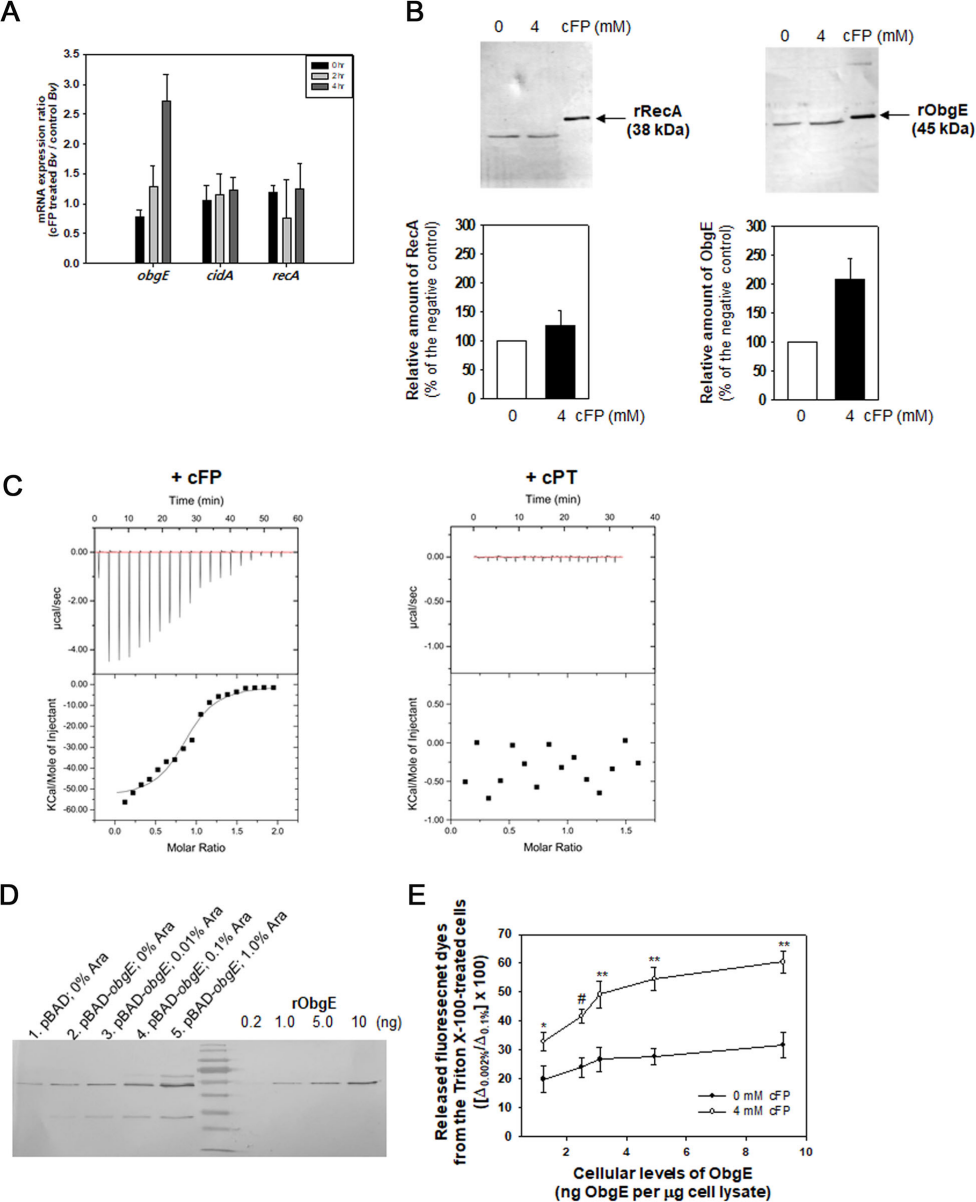
Role of ObgE in cFP-mediated change in the membrane permeability of *B. vulgatus*. **A** Transcript levels of *obgE, recA,* and *cidA* in cFP*-*treated *B. vulgatus*. The total RNAs were extracted from *B. vulgatus* cells treated with 4 mM cFP for 0, 2, and 4 h. Then, cDNAs were produced through the reverse transcriptase reaction and subjected to quantitative PCR using primer sets specific to *obgE, recA,* or *cidA* (Additional file: Table S3). The transcript contents of each gene were normalized with the *gap* transcript levels. The ratios of normalized contents in cFP-treated cells to those in the control cells were plotted. **B** Protein levels of ObgE and RecA in cFP*-*treated *B. vulgatus*. Fifteen micrograms of protein lysates of *B. vulgatus*, which had been exposed to 0 or 4 mM cFP for 4 h, were fractionated in SDS-PAGE and subjected to western blotting analysis using anti-ObgE and anti-RecA polyclonal antibodies. As a positive control, rRecA (38 kDa) and rObgE (45 kDa) were included in the blots. Densitometric readings of the bands corresponding to each protein in the control and cFP-treated cells were plotted. **C** Specific binding of cFP to ObgE. Isothermal titration calorimetry (ITC) experiments were performed by titrating the recombinant ObgE (40 μM) with 400 μM cFP at 25°C. As a control, rObgE was titrated with 400 μM cPT under the same condition for ITC. **D** Differential expression of ObgE in *B. vulgatus* cells. Freshly grown *B. vulgatus* carrying the vector plasmid (pBAD/*Myc*-His B) or pBAD-*obgE*_Bv_ was added with various concentrations of *L*-arabinose ranging from 0% to 1.0% to differentially induce *obgE* expression. Upon 1 h induction, bacterial lysates (2 μg) were subjected to western blotting along with the known concentrations (from 0.2 to 10 ng) of rObgE to estimate the cellular contents of ObgE. **E** Effect of the cellular levels of ObgE on membrane permeability. The same cells prepared in Fig. 8D were exposed to 4 or 0 mM cFP for 6 h then mixed with 0.4 μΜ DiSC3(5). As described in Fig. 7, the values of released DiSC3(5) from 0.002% (Δ_0.002%_) and 0.1% Triton X-100-treated cells (Δ_0.1%_) were estimated. The ratios ([Δ_0.002%_/Δ_0.1%_] × 100) were plotted against the cellular contents of ObgE. shown in Fig. 8D. *P*-values for the comparison of 4 mM cFP-treatment with no cFP-treatment are indicated (Student’s *t*-test; ns, not significant; *0.001 < *P* < 0.01; **P < 0.001; ^#^no *p* value determined)

Some factors, such as RecA, CidA, and ObgE, have been found to regulate the integrity of bacterial membranes under specific conditions [29–32]. The genomes of *B. vulgatus* have the open reading frames (ORFs) homologous to these genes, though their functions have not yet been studied. To screen the factor(s) involving the cFP-mediated membrane disruption, the cellular abundance of *obgE*, *cidA*, and *recA* transcripts and proteins were monitored in *B. vulgatus* cells incubated under the conditions in the absence or presence of cFP. Transcript levels of the *obgE*, of which contents were normalized with the *gap* transcript levels in the same cells, were increased as the exposure time was extended (about three-fold increase in the 4 h incubation compared to 0 h incubation), while those of *cidA* and *recA* were not altered up to 4 h incubation period (Fig. 8A). Protein levels in cells were examined using specific polyclonal antibodies against recombinant ObgE and RecA of *B. vulgatus* (Fig. 8B). Due to no apparent induction of rCidA from the overexpression vector carrying the *cidA* gene, an experiment to compare the cellular CidA levels was not successful. In addition to the increase in the *obgE* transcript, the protein content of ObgE increased in the *B. vulgatus* cells exposed to cFP. These results lead us to speculate the possible involvement of ObgE in the observed membrane disruption in *B. vulgatus* exposed to cFP.

To elucidate the specific interaction of ObgE with cFP, the isothermal titration calorimetry (ITC) experiments were performed using nucleotide-free rObgE, as described in “Methods.” In total, 40 μM of rObgE was titrated at 25 °C with a cFP stock solution of 400 μM (Fig. 8C). The equilibrium dissociation constant (K_D_) and binding stoichiometry (*N*) for the interaction between rObgE and cFP were 1.8 ± 0.35 μM and 0.78 ± 0.11, respectively. In contrast, the titration of rObgE with 400 μM of another cyclic dipeptide, cPT did not show any signature for the inter-molecular interaction.

Since the *B. vulgatus* ObgE specifically interacted with cFP and its cellular contents increased in cFP-treated cells, the role of ObgE in membrane disruption was examined. It has been previously reported that a trial of *obgE* gene deletion was not successful in many bacterial species [33]. Therefore, we have constructed a cell enabling to overexpress ObgE in response to the concentration of arabinose, instead to attempt to construct an *obgE* deletion mutant of *B. vulgatus*. Differential expression of ObgE was achieved using *B. vulgatus* MGM001 harboring the *obgE*_Bv_ gene in a vector, pBAD/*Myc*-His B (pBAD-*obgE*_Bv_), which was added with *L*-arabinose at concentrations ranging from 0 to 1.0%. For quantitative analysis, each cell lysate was subjected to western blotting along with the known concentrations of rObgE (Fig. 8D). Cellular contents of ObgE were estimated by extrapolating the densitometric readings of the ObgE bands derived from cell lysates to the regression line derived from those of the known concentrations of rObgE (from 0.2 to 10 ng). The estimated cellular contents of ObgE were increased from 1.3 to 2.5 ng ObgE per μg of cell lysate by carrying pBAD-*obgE*_Bv_, and those of *B. vulgatus* cells harboring pBAD-*obgE*_Bv_ were further increased to 3.1, 4.9, and 9.3 ng ObgE per μg of cell lysate if cells were incubated with 0.01%, 0.1%, and 1.0% arabinose, respectively.

Then, we measured the membrane integrity in *B. vulgatus* cells having different levels of ObgE. The same cells prepared for the experiments shown in Fig. 8D were exposed to 4 or 0 mM cFP for 6 h and then mixed with 0.4 μΜ DiSC3(5) for 60 min. As described above, the values of released DiSC3(5) from 0.002% Triton X-100-treated cells (Δ_0.002%_) were estimated and normalized with the total DiSC3(5) initially incorporated into cells (Δ_0.1%_). The ratios ([Δ_0.002%_/Δ_0.1%_] × 100) shown in Fig. 8E revealed that the increase in cellular ObgE resulted in the increased release of the dyes upon treatment of 0.002% Triton X-100 in an ObgE concentration-dependent manner (closed circles). Furthermore, the exogenous addition of 4 mM cFP resulted in 1.7~2.0 times greater release of DiSC3(5) (open circles) than the each control cells without cFP treatment. These differences were significant with *P*-values less than 0.01 (Student’s *t*-test).

### Alteration of mouse lethality by *V. vulnificus* via changes in *B. vulgatus* abundance in mouse guts

**Fig. 9:**
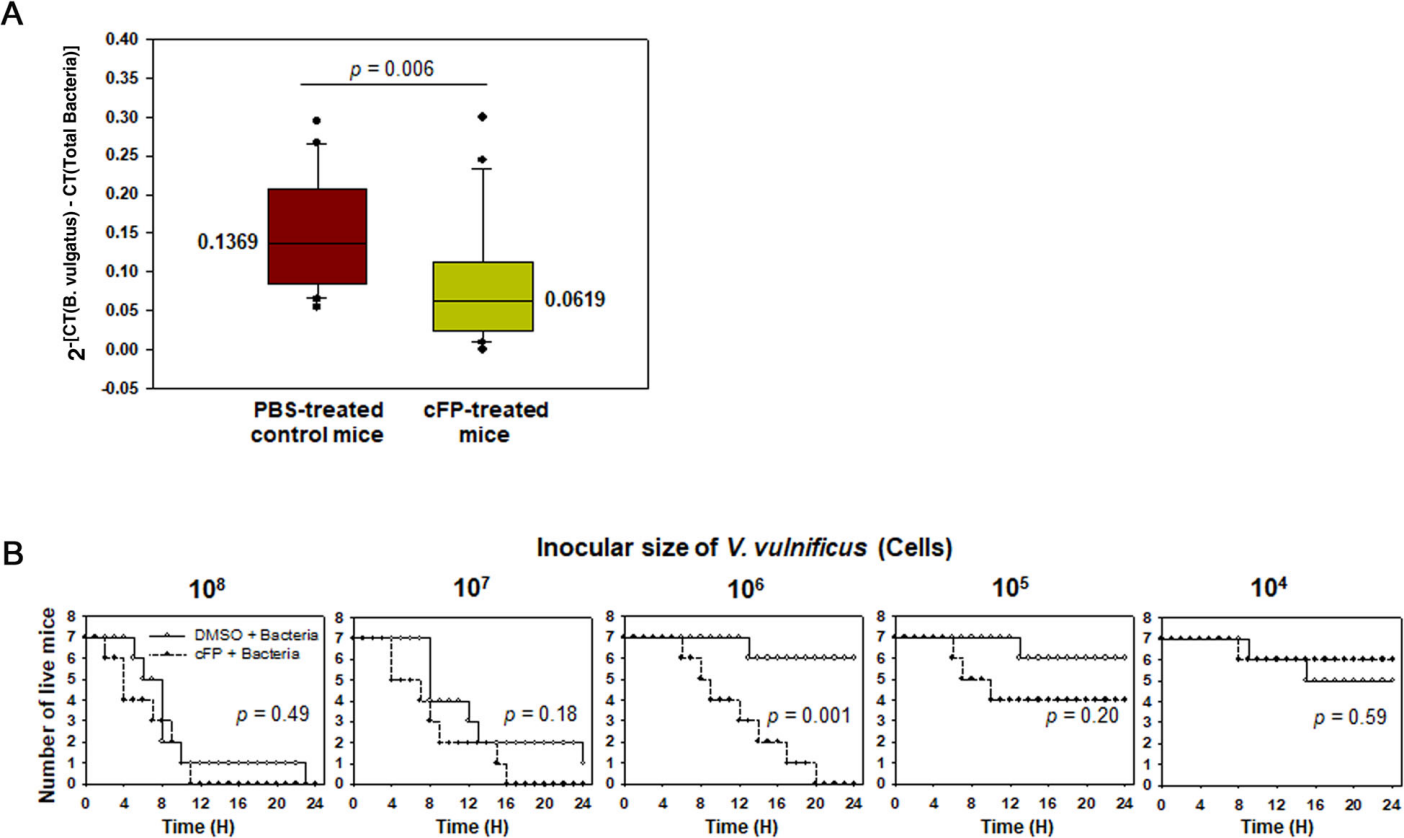
In vivo effects of cFP in mice. **A** Effect of cFP on the abundance of *B. vulgatus* in fecal samples. The fecal samples were collected from four-week-old female mice orally injected with cFP or PBS (*n* = 20 mice per each set). The total DNA extracted from each fecal sample was subjected to q-PCR using a primer set specific to *B. vulgatus* 16S rDNA and the universal primer set for eubacterial 16S rDNA. The relative abundance of *B. vulgatus* 16S rDNA was normalized with the abundance of the total bacterial 16S rDNAs, and presented by the values of 2^−[CT(*B.vulgatus*) − CT(Total Bacteria)]^ with their median numbers. Statistical analysis was performed using the Student’s *t*-test and the resulting *P*-value was provided. **B** Effect of cFP on mouse lethality caused by *V. vulnificus*. Four-week-old female mice were orally injected with cFP as described above, then infected with various doses of *V. vulnificus*, ranging from 10^4^ to 10^8^ cells (*n* = 7 mice per each treatment) (open symbol). The same experiments were performed using the control mice, which had not been injected with cFP (closed symbol). The surviving mice were enumerated for 24 h, and their numbers were plotted against incubation time. Statistical analyses were performed using the log-rank test and the resulting *P*-values were provided

The introduction of *V. vulnificus* resulted in the reduction of *B. vulgatus* levels in the fecal samples and this reduced composition was postulated to be mediated by cFP secreted by infecting *V. vulnificus*. Therefore, the effect of the exogenous addition of cFP on the abundance of *B. vulgatus* in mouse fecal samples was examined. The fecal samples were collected from 4-week-old female mice orally injected with cFP at a concentration of 110 μg cFP per gram of mouse. The total DNA extracted from each fecal sample was subjected to q-PCR using a primer set specific to the 16S rDNA of *B. vulgatus* (Bv-F and Bv-R [34];) or the universal primer set for the eubacterial 16S rDNA (785F and 907R [35];). The relative abundance of *B. vulgatus* 16S rDNA was normalized by the abundance of the total 16S rDNAs derived from PCR using the universal primer set. The medians of estimated values of 2^−[CT(*B.vulgatus*) − CT(Total Bacteria)]^ were 0.1369 (± 0.0748) and 0.0619 (± 0.0766) in the PBS-treated control (*n* = 20 mice) and cFP-treated mice (*n* = 20 mice), respectively (Fig. 9A). Therefore, the levels of *B. vulgatus* decreased approximately 2.2-fold in mice injected with cFP (*P* = 0.006, two-sided Student’s *t*-test).

Next, we investigated whether the mice containing decreased levels of *B. vulgatus* showed a different susceptibility to infection by *V. vulnificus*. Mice were orally injected with cFP and they were infected at 12 h post-injection of cFP with various doses of *V. vulnificus*, ranging from 10^4^ to 10^8^ cells (*n* = 7 mice per each treatment) (open symbol). The same experiments were performed using the control mice that had been injected with cFP-free DMSO cells (*n* = 7 mice per each treatment) (closed symbol) (Fig. 9B). When infected with the highest dose of *V. vulnificus* (i.e., 10^8^ cells), mice under both conditions died equally and quickly within 24 h. Similarly, the mice infected with the lowest dose of *V. vulnificus* (i.e., 10^4^ cells) showed similar patterns of survival, regardless of the exogenous addition of cFP. However, the number of dead mice was higher in the cFP-treated sets of mice when they were preinjected with cFP then infected with 10^5^–10^7^ cells of *V. vulnificus*. In cases of infection with 10^6^ cells of *V. vulnificus*, the difference in survival was significant, with a *P*-value of 0.001 (log-rank test).

## Discussion

Cyclic dipeptides, formed by combining two amino acids through the reactions involving non-ribosomal peptide synthetases or cyclodipeptide synthases, are produced by a wide range of microorganisms [36]. Among them, pathogenic *Vibrio* species have been shown to produce cFP and secrete it in ambient environments at high concentrations [24]. The extracellular cFP serves as a signal molecule to regulate the expression of their virulence factors. For example, cFP induces the expression of *ompU* (encoding a major outer membrane protein) and *katG* (encoding the hydroperoxidase I) via a regulatory pathway composed of ToxR and LeuO in *V. vulnificus* [37–39], but it downregulates the expression of *ctx* (encoding Cholera toxin) and *tcpPH* (encoding toxin-coregulated pilus) via the ToxR-LeuO-AphA pathway in *V. cholerae* [40]. In addition to the role of cFP as a signal to bacteria themselves, cFP has the activities to their host cells by inhibiting the innate immune responses mediated by RIG-I and NF-κB [27, 41] and inducing the damage of mammalian DNA via increased ROS [42]. Therefore, the spectrum of roles of cFP in *Vibrio* pathogenicity has been broadened to be one of their virulence factors. Furthermore, the regulatory role of cFP at another level of *V. vulnificus*-host interactions was identified in this study. cFP was able to induce the cell death of *B. vulgatus*, a major constituent of the gut microbiota (Figs. 4 and 5). This finding is very interesting since a signal used by a pathogen to regulate its virulence factors is also utilized to change the gut environments to favor its pathogenicity by specifically transducing the signal to a group of gut commensals and removing these bacteria from the guts.

The survival of three strains of *B. vulgatus* in the presence of *V. vulnificus* and cFP was tested in this study: ATCC8482 from human feces; DSM28735 from a mouse caecum; and MGM001 isolated from a mouse feces (Figs. 2 and 4). A decrease in *B. vulgatus* CFUs in incubations with cFP was evidenced to be due to the actual death of bacterial cells, upon fluorescence microscopy observation of the *B. vulgatus* cells stained with the live/dead-indicating dyes (Fig. 5A and B). cFP-mediated death of *B. vulgatus* was accompanied with alterations in membrane integrity, which was discernible in the images taken under the electron microscopes (Fig. 5C–F). Disruptive changes in their membranes were determined using a fluorescent dye DiSC3(5), which was incorporated into the bacterial membranes. Upon exposure to a membrane permeabilizing agent at its EC_50_ (e.g., 0.002% Triton X-100), the released DiSC3(5) was monitored by fluorometry, showing that significantly more dyes were detected from the cFP-treated cells than the control (Fig. 7B). It suggests that cFP treatment made *B. vulgatus* membranes more susceptible to membrane permeabilizing stress. However, when cells were treated with other cyclic dipeptides consisting of hydrophobic amino acids (e.g., cPT and cPV) or a linear dipeptide of FP, the amounts of released DiSC3(5) upon exposure to 0.002% Triton X-100 were almost the same as those of the control (Additional file: Figure S2). Therefore, the effect of cFP on changing the membrane integrity is highly specific to *B. vulgatus*.

In addition to *B. vulgatus*, *P. goldsteinii* belonging to Bacteroidetes also showed the similar response to *V. vulnificus* and cFP (Fig. 6). Microbiome composition data derived from two sets of experiments with the mice infected with *V. vulnificus* (Additional file: Table S1) showed a decrease in the abundance of *P. goldsteinii* in the *V. vulnificus*-fed mice, compared to the control PBS-fed mice (Mann-Whitney *U* test, *P* = 0.050): from 1.81% (± 0.96%) to 0.67% (± 0.18%) in experiment 1; and 0.91% (± 0.41%) to 0.35% (± 0.003%) in experiment 2. Furthermore, the fecal samples collected from mice orally injected with cFP, as shown in Fig. 9A, contained significantly less copies of *P. goldsteinii*-specific 16S rRNA genes than the control mice (*P* = 0.038, two-sided Student’s *t*-test) (Additional file: Figure S3). In contrast, the abundance of *L. reuteri*-specific 16S rRNA genes was not significantly altered in the control and cFP-fed mice (*P* = 0.596, two-sided Student’s *t*-test). However, it is worth note that the abundance of *B. caccae* in mouse guts was not significantly affected by *V. vulnificus* infection (Mann-Whitney *U* test, *P* = 0.221) (Additional file: Table S1), and *B. fragilis* and *B. ovatus* exhibited high susceptibility to multiple dipeptides including cFP [43]. Therefore, it is speculated that cFP is a specific driver in the *V. vulnificus*-fed mice to reduce the abundance of some species of *Bacteroides* and *Parabacteroides*.

The specific interaction of cFP to *B. vulgatus* in inducing increased susceptibility to a membrane permeabilizing agent further suggests the presence of a specific sensor for cFP, which is able to induce cell death via membrane disruption. Previous studies have found that some bacterial proteins, e.g., RecA, CidA, and ObgE, play roles in regulating the permeabilization or depolarization of membranes [29–31]. Among them, ObgE of *B. vulgatus* responded to exogenously added cFP. The role of ObgE in cFP-mediated membrane disruption and subsequent cell death was not able to be confirmed with an *obgE*-deleted mutant of *B. vulgatus*, since the trials of *obgE* gene deletion have not been successful in diverse species of bacteria [33]. Increase in membrane disruption via ObgE was instead evidenced in the ObgE-overexpressing *B. vulgatus*, where the susceptibility degrees towards membrane-permeabilizing stress(es) were proportional to the cellular levels of ObgE (Fig. 8D and E). ObgE is a conserved GTPase among a wide-range of bacterial species, and it is reported to be essential for their viability by mediating the stringent response, ribosomal assembly, DNA replication and morphological differentiations including a sporulation process in spore-forming bacteria [31, 44, 45]. ObgE has been proposed to change the membrane potential of *E. coli* and *P. aeruginosa* by activating the expression of a toxin-antitoxin module of HokB and SokB under stringent conditions [31]. However, both *hokB* and *sokB* ORFs are not present in the genomes of *B. vulgatus*. Interestingly, a substitution of an amino acid residue in the G domain of *E. coli*’s ObgE (ObgE*) resulted in the induction of programmed cell death via an unknown mechanism [32, 46]. It is speculated that the ObgE of *B. vulgatus* may have activity similar to the ObgE*. Furthermore, since the *B. vulgatus* ObgE is specifically bound by cFP at a ratio of 1:1 (Fig. 8C), formation of the cFP-ObgE complex increases this speculative activity inducing membrane disruption and subsequent cell death. *In silico* analysis of various ObgE homologs revealed that the ObgEs of *B. vulgatus* and *P. goldsteinii* have a conserved stretch of amino acids named by the DNA Pol Phi domain [47], which is overlapped with the fifth GTP-binding motif (G5) and the C-terminal domain (Additional file: Figure S4). In contrast, the ObgEs of other gut commensals including *B. caccae* and *Lactobacillus* spp. do not show the presence of this domain at their C-terminal regions. Future studies should identify the role of the conserved domain in the *B. vulgatus* ObgE in interacting with cFP and/or GTP, and the signal transducing mechanism underlying the membrane disruption via ObgE complexed with cFP.

Maintenance of normal microflora in GI tracts is important in the host response to pathogens [48]. Therefore, the conditions of an imbalance in the microbiota, such as reduced microbial diversity or fluctuated abundance of the obligate and facultative anaerobes in the gut microbiomes, resulted in high susceptibility to pathogenic colonization [48]. An obligate anaerobic Gram-negative bacterium, *B. vulgatus*, is a predominant microorganism in the mammalian GI tracts and the core gut commensal in healthy humans [49, 50]. Although *B. vulgatus* shows the ability to efficiently metabolize some nutrients in the guts and plays roles in preventing inflammation and protecting the LPS-associating infections [51–53], it remains unclear why *B. vulgatus* is abundantly residing in the intestinal tracts and how it provides benefits to the host. In the present study, mice with decreased abundance of *B. vulgatus* in their guts due to pretreatment with cFP showed increased lethality when infected by some doses of *V. vulnificus* (Fig. 9A and B). These results lead us to speculate that this foodborne pathogen would increase its pathogenic potential by initially antagonizing the preexisting mouse gut commensals, which could strongly provide colonization barriers on the mucus layers [10]. Then, the pathogens could increase their accessibility to the gut epithelial cells and their success in causing fatal septicemia.

## Conclusions

This study demonstrates a clear antagonistic effect of a foodborne pathogen, *V. vulnificus*, on a gut resident, *B. vulgatus*. This bacterial cell-to-cell interaction was specifically mediated by cFP and ObgE and caused *B. vulgatus* cells to be highly sensitive to the membrane-disrupting stress(es). The cFP-mediated death of *B. vulgatus* was also observed in vivo; thus, a signal molecule of *V. vulnificus*, cFP, plays a role in enhancing its pathogenic potential in the host by modulating the abundance of the predominant species among gut commensals.

## Methods

### Bacterial culture conditions

All bacterial strains and plasmids used in this study are listed in the Additional file: Table S2. *Vibrio* strains were grown at 30~37 °C in LBS (Luria-Bertani [LB] medium containing NaCl at a final concentration of 2.5% [w/v]) under aerobic conditions, unless otherwise mentioned. *B. vulgatus* and *P. goldsteinii* were grown in RCM (Reinforced Clostridial medium; BD Difco) in an anaerobic chamber (Whitley DG250 Anaerobic Workstation) adjusted to 37 °C. *Lactobacillus* strains were grown at 37 °C in MRS (De Man, Rogosa and Sharpe; BD Difco) media under anaerobic conditions. For coculturing the cells of *V. vulnificus* and *B. vulgatus* on a single agar plate, each cell suspension was spotted on RCMS (RCM supplemented with 2.5% sodium chloride), and incubated in a 37 °C anaerobic chamber. *E. coli* used for plasmid DNA preparation and conjugal transfer were grown at 37 °C in LB medium supplemented with appropriate antibiotics. Antibiotics were used at the following concentrations: for *E. coli*, ampicillin at 100 μg/ml and chloramphenicol at 20 μg/ml; for *B. vulgatus*, ampicillin at 10 μg/ml; and for *V. vulnificus,* chloramphenicol at 2 μg/ml.

### Animal experiments

Experiments were performed using 4- or 7-week-old female ICR mice (CrljOri:CD1[ICR], OrientBio, Korea). Mice were maintained at 23 °C and 50% humidity under the condition of a light/dark cycle of 12/12 h, and had free access to water and food (LabDiet 5L79, OrientBio, Korea). After acclimation to these laboratory conditions for 2 days, mice were challenged to the specific treatments for each experiment. During the whole experiment procedures, mice received humane care in accordance with the institutional guidelines of Sogang University and the legal requirements (permit numbers, IACUCSGU2015_03 and IACUCSGU2019_01). All efforts were made to minimize animals suffering and to reduce the number of mice used.

### Fecal sample collection and microbiome analysis

Seven-week-old female ICR mice were intraperitoneally injected with iron dextran (30 μg per gram of mouse weight) and intragastrically injected with 50 μl of 8.5% (w/v) sodium bicarbonate. After 12 h, each mouse was intragastrically infected with 2.0 × 10^9^ *V. vulnificus* cells suspended in phosphate buffered saline (PBS) using an oral gavage needle, then placed in a separate cage. As a control, mice were treated with *V. vulnificus*-free PBS (two mice per set of the infection experiment). Fecal samples were collected at every hour if they were present in the cages housing an individual mouse for 20 h. To compensate the probable variations among the batches of mice, two independent sets of infection experiments were performed, and the five and four fecal samples were retrieved from the dead and healthy mice, respectively.

The total DNAs in the samples were extracted using FastDNA® Spin Kit (MPbio), and the amounts of DNA were quantified by Epoch™ Spectrophotometer (BioTek). DNA samples from experiments 1 and 2 were subjected to PCR to amplify the V1 to V3 regions of 16S rDNA using the barcoded primers of 9F and 541R [54]. Products purified through the QIAquick PCR purification kit (Qiagen) were pooled together and the short non-target products were removed through the Ampure beads kit (Agencourt Bioscience). Quality control (QC) of the amplified DNA was assessed on a Bioanalyzer 2100 (Agilent) using a DNA 7500 Chip, and pyrosequencing was carried out with a GS Junior Sequencing system (Roche). Sequencing experiments were performed by ChunLab Inc (Korea) and the sequence data analyses were processed [55]. The obtained reads from the different samples were sorted by unique barcodes in each PCR product. The sequences corresponding to the barcode, linker, and primers were removed from the original sequencing reads. Any read containing multiple ambiguous nucleotides, low-quality score (average score < 25), or shorter than 300 bp were discarded. Potential chimera sequences, which were detected by the bellerophone method [56] were also removed. The taxonomic classification of each read derived from the experiments 1 and 2 was assigned against the EzTaxon-e database deposited in EzBioCloud [57], which contains 16S rRNA gene sequence of type strains that have valid published names and representative species-level phylotypes of either cultured or uncultured entries in the GenBank database with complete hierarchical taxonomic classification from the phylum to the species. Statistical analysis of the taxonomically classified sequencing data was processed using the EzBioClaud. Principal coordinates analysis (PCoA) of beta diversity metrics based on Jensen-Shannon distances was estimated and visualized using EzBioCloud. The difference of microbial communities among the fecal samples were calculated using permutational multivariate analysis of variance (PERMANOVA) [58].

### Isolation of mouse gut microorganisms

To obtain pure cultures of bacteria in the mouse guts, the fecal samples collected from seven-week-old female ICR mice were resuspended in PBS. The resuspension was briefly centrifuged to eliminate fecal debris and the supernatants were retrieved. Diluted suspensions were spread onto GAM (Gifu Anaerobic medium; BD Difco), RCM, or MRS agar plates and incubated at 37°C for 24~48 h under anaerobic conditions. Bacterial colonies were processed to amplify their 16S rDNA fragments using the 27F and 1492R primers and the purified PCR products were subjected to DNA sequencing analysis using the 785F and 907R primers (Macrogen, Korea). Nine bacterial isolates showed more than 97% identity to the corresponding regions of 16S rDNAs of *B. vulgatus*, *P. goldsteinii*, *L. johnsonii*, *L. reuteri*, *L. murinus*, or *L intestinalis*, and were named by MGM001 to 009 (Additional file: Table S2). DNA sequences of 16S rRNAs were deposited to the GenBank with accession numbers of MT764994 to MT765002.

### Survival tests of *B. vulgatus*

Freshly grown cells of *B. vulgatus* were washed twice and resuspended in the M9-salt minimal medium [59] to contain cells at an OD_595 nm_ of 1.0. Then, the cell suspensions were added by various bacterial cells, their spent media (SM), or chemical compounds, and were incubated at 37 °C. All procedures were conducted in an anaerobic chamber. At the designated incubation time for each experiment, the mixtures were serially diluted with M9-salt minimal medium and the resultant aliquots were spread on the RCM agar plates. After 48 h of incubation in an anaerobic chamber, the viable *B. vulgatus* cells were counted. For coculture experiments, freshly grown *Vibrio* species (*V. vulnificus*, *V. cholerae*, and *V. parahaemolyticus*) and *E. coli* (a strain of S17-1 λ*pir*) were harvested, washed twice, and resuspended in the M9-salt minimal medium to contain appropriate cell densities for each experiment. In case of coincubation with *V. parahaemolyticus*, which was able to grow on the RCM plate under anaerobic conditions, its CFUs were also enumerated by spreading the same samples on the TCBS (thiosulfate-citrate-bile salt-sucrose) agar plates under aerobic conditions and then subtracted from the total CFUs on the RCM plates incubated under anaerobic conditions. SM of *V. vulnificus* were prepared using 0.45 μm-pore-sized filter to sterilize the supernatants of the anaerobic culture in LBS. Solutions of organic acids (pyruvate, formate, lactate, and acetate [Sigma Aldrich]), cyclic dipeptides (cFP, cPV, and cPT [Bachem Inc.]), and a dipeptide (FP [Sigma Aldrich]) were prepared in the deionized water or DMSO.

### Microscopy observation

*B. vulgatus* MGM001 cells treated with cFP (0, 1, 2, and 4 mM) for 6 h were stained with a Live/Dead cell double staining kit (Sigma) and observed under a confocal laser scanning microscope (LSM700, Carl Zeiss). The viable cells were observed at an excitation wavelength of 488 nm and an emission wavelength of 515 nm, and the dead cells were observed at an excitation wavelength of 535 nm and an emission wavelength of 617 nm. The numbers of viable and dead cells, which were shown as green and red cells, respectively, were counted and their average numbers per microscope field were obtained through at least eight observations.

MGM001 cells treated with cFP (0 and 4 mM) for 6 h were processed for SEM analysis. Bacterial cells were fixed with 2% glutaraldehyde-paraformaldehyde in 0.1 M phosphate buffer (PB, pH 7.4) for 24 h and then with 1% OsO4 dissolved in 0.1 M PB for 1.5 h. Fixed cells were dehydrated through an eight-stepped ascending gradual series of ethanol (from 50% to 100%), infiltrated with isoamyl acetate, and placed in the Critical Point Dryer (Leica EM CPD300). The resultant samples were coated with 5 nm-thick Pt by an Ion Coater (Leica EM ACE600) and examined under Merlin^TM^ FE-SEM (Carl Zeiss) operating at 2 kV.

*B. vulgatus* (MGM001, ATCC8482, and DSM28735) and *P. goldsteinii* (MGM002) exposed to cFP (0 and 4 mM) for 6 h were processed for TEM analysis. The cells fixed by treating with 2% glutaraldehyde-paraformaldehyde (for 12 h) and 1% OsO4 (for 2 h) were dehydrated as described above, and infiltrated with propylene oxide. Then, samples were embedded using the Poly/Bed 812 kit (Polysciences) and treated at 65 °C for 24 h in an electron microscope oven (TD-700, Dosaka). Polymerized samples were sectioned (200–250 nm) using ultramicrotome, then further thin-sectioned (70 nm) using Leica EM UC-7 (Leica Microsystems) and a diamond knife (Diatome). The sections were double-stained with 6% uranyl acetate and lead citrate, transferred onto the copper-nickel grids (Electron Microscopy Science), and examined under JEM-1011 TEM (JEOL).

### Membrane permeability assay

The effect of cFP on bacterial membrane permeability was determined using a membrane potential-sensitive fluorescent dye, DiSC3(5) (Sigma). Freshly grown *B. vulgatus* cells were washed twice and resuspended in the M9-salt minimal medium to contain cells at an OD_595 nm_ of 1.0. Four millimolar cFPs dissolved in DMSO were added to the cell suspension. The same volume of DMSO was added as a control. At 6 h of exposure to cFP or DMSO, cells were washed twice with 5 mM HEPES buffer (pH 7.4) and then treated for 1 h with 0.4 μM of DiSC3(5) dissolved in 5 mM HEPES including 100 mM KCl. After unincorporated dyes were discarded, the cells were resuspended in the same volume of 5 mM HEPES. Aliquots (100 μl) of the dye-associated cells were placed in 96-well black polystyrene microplates (SPL life science). RFUs were measured in a fluorometer (EnSpire Multimode Plate Reader, PerkinElmer), for which the temperature was set at 30 °C and wavelengths of 622 and 670 nm, for excitation and emission, respectively. At 0.5 h in a fluorometer, Triton X-100 (0.002% or 0.1%) was added to each well and the RFUs were continuously measured for another 0.5 h. The released fluorescence from the Triton X-100-treated *B. vulgatus* cells was obtained by subtracting the values of basal RFU (averaged RFUs for 10 min before treatment of Triton X-100) from the maximal RFU (averaged RFUs for 10 min after treatment of Triton X-100).

### Construction of *B. vulgatus* expressing *obgE* in response to arabinose

A 1185-bp DNA fragment containing the *obgE* gene of *B. vulgatus*, which was amplified using the primers of ObgE_pBAD-F and ObgE_pBAD-R (Additional file: Table S3), was digested with NcoI and KpnI and ligated into a vector plasmid pBAD/Myc-His B. The resultant pBAD-*obgE*_Bv_ was transformed into *E. coli* S17-1λ*pir* then transferred to *B. vulgatus* MGM001, which is naturally sensitive to ampicillin, via conjugation between two species of bacteria. *B. vulgatus* harboring pBAD-*obgE*_Bv_ was selected by spreading aliquots of the conjugate mixture on the RCM agar plate supplemented with 10 μg/ml ampicillin and incubating in an anaerobic chamber.

### Western blotting analysis using anti-ObgE and anti-RecA polyclonal antibodies

Two oligonucleotides, Bv_obgE-over F and Bv_obgE-over R (Additional file: Table S3), were used to amplify a 1185 bp DNA fragment containing *obgE* ORF from the genomic DNA of *B. vulgatus* MGM001. Amplified DNA fragments were digested with BamHI and SalI, and cloned into pET28b to generate pET28b-*obgE*. In the same way, DNA fragments containing *recA* ORF (1064 bp) amplified using Bv_recA-over F and Bv_recA-over R (Additional file: Table S3) were cloned into pET28b to generate pET28b-*recA*. Each recombinant protein was overexpressed in *E. coli* BL21 by adding 1 mM of IPTG and purified using a Ni-NTA affinity column, as directed by the manufacturer (Qiagen). The purified recombinant proteins were used to generate polyclonal antibodies by the three immunizations of ICR mice (CrljOri:CD1[ICR]) (100 μg of recombinant proteins per immunization) at 3-week intervals.

To prepare the total cell lysates of *B. vulgatus*, cells were resuspended in Tris-buffered saline with Tween 20 (TBST; 150 mM NaCl, 50 mM Tris-HCl, and 0.1% Tween 20) and lysed by sonication. Then, 20 μg of each cell lysate was loaded onto a 12% SDS-PAGE, run for 1 h at 120 V, and transferred to Hybond P membrane (Amersham). Membranes were blocked with 5% skim milk in TBST for 30 min and incubated overnight at 4°C with each polyclonal antibody (1:5000, [vol/vol]), followed by alkaline phosphatase-conjugated rabbit anti-mouse IgG (1:5000, [vol/vol]). Immunoreactive bands were visualized using the NBT-BCIP system (Promega).

### Isothermal titration calorimetry

Binding characteristics of rObgE to cFP were analyzed using the MicroCal iTC200 system (Malvern Panalytical) with a reference power of 10 μcal/s. The thermodynamic parameters upon titration of rObgE (40 μM, nucleotide-free) with a solution of 400 μM cFP were measured at 25 °C in a buffer consisting of 20 mM HEPES (pH 7.5), 150 mM NaCl, 5 mM MgCl_2_, and 1 mM β-mercaptoethanol. The nucleotide-free rObgE was prepared by treating with calf intestine alkaline phosphatase (Promega) and passing a gel filtration AKTA-FPLC system (Superdex 200, GE Healthcare) at a flow rate of 0.3 ml/min in a buffer containing 20 mM HEPES (pH 7.5), 150 mM NaCl, and 5 mM MgCl_2_ as previously described [60]. rObgE in the eluted fractions (1 ml) was concentrated using Amicon® Ultra-15 10K (Millipore) and its buffer was exchanged with that used for an ITC assay. To obtain the equilibrium dissociation constant (K_D_) and binding stoichiometry (*N*), the data were fitted to a single binding site model using the standard Marquardt non-linear regression method as provided from the MicroCal ITC-Origin Analysis (Malvern Panalytical).

### Quantitative PCR

Fecal samples were collected from the mice fed with PBS or cFP for 6 and 24 h, and their bacterial DNAs were extracted using ZymoBIOMICS^TM^ DNA miniprep kit (ZYMO research). Using 2.5 ng of DNA in each sample, qPCR was run with the primer set specific to 16S rDNA of *B. vulgatus* (Bv-F and Bv-R; 34) and the universal primer set of 785F and 907R [35] using a LightCycler 480 II (Roche Life Science). Threshold cycle (CT) values representing the relative quantities of the *B. vulgatus*-specific 16S rDNA and the total bacterial 16S rDNAs in each sample were obtained and the values of 2^−[CT(*B.vulgatus*) − CT(Total Bacteria)]^ were calculated by the 2^−ΔΔCT^ method [61]. The primers used in this assay are listed in the Additional file: Table S3.

### Mouse lethality assay

To examine the mortality of the mice infected with *V. vulnificus* in the presence of cFP, four-week-old female ICR mice (CrljOri:CD1[ICR]), which have been starved for 12 h, were intragastrically treated with cFP (110 μg cFP per gram of mouse) for 12 h prior to *V. vulnificus* infection. Before bacterial infection, each mouse was intraperitoneally injected with iron dextran (30 μg per gram of mice weight) and intragastrically injected with 8.5% [w/vol.] sodium bicarbonate (50 μl). Various concentrations of freshly grown *V. vulnificus* were resuspended in PBS and injected intragastrically into seven mice per set. Since cFP was dissolved in PBS mixed with 1% DMSO, the same volume of buffer was injected intragastrically as a control. The number of dead mice was counted for 24 h.

### Statistical analyses

Results were expressed as the mean ± standard deviation (SD) or median ± SD in the three independent experiments. Statistical analysis was performed using Student’s *t*-test (SYSTAT program, SigmaPlot version 9, Systat Software Inc.). A log-rank test (http://bioinf.wehi.edu.au/software/russell/logrank/) was performed to estimate statistical significance of the mouse mortality results. Differences were considered significant if *P*-values were < 0.01. *P*-values were presented in the corresponding figures, or their significance was indicated by * or **, when 0.001 < *P* ≤ 0.01 or *P* < 0.001, respectively.

## Supplementary Information

**Additional file 1.**

## Data Availability

Amplicon data for the phylum Bacteroidetes are in the Additional file: Table S1; information for the bacterial strains, plasmids, and oligonucleotide primers are listed in the Additional file: Tables S2 and S3; the 16S rRNA sequences for the MGM isolates were deposited to the GenBank with accession numbers of MT764994 to MT765002.

## Abbreviations

cFP: Cyclo-(phenylalanine-proline)
CFU: Colony-forming unit
cPT: Cyclo-(proline-threonine)
cPV: Cyclo-(proline-valine)
DiSC3(5): 3,3′-Dipropylthiadicarbocyanine iodide
ITC: Isothermal titration calorimetry
RFU: Relative fluorescence unit
SM: Spent medium
