# Supplementary material for: Vibrio vulnificus induces the death of a major bacterial species in the mouse gut via cyclo-Phe-Pro
Source: Microbiome. 2021 Jul 20;9:161. doi: 10.1186/s40168-021-01095-w (PMC8293591; doi:10.1186/s40168-021-01095-w)
Supplement: Supplementary file 2 — Additional file 1. [file 40168_2021_1095_MOESM2_ESM.pdf]

***Vibrio vulnificus* Induces the Death of a Major Bacterial Species in the Mouse Gut via Cyclo-Phe-Pro**

Jeong-A Kim[1], Bo-Ram Jang[1], Yu-Ra Kim, You-Chul Jung, Kun-Soo Kim, and Kyu-Ho Lee*

Department of Life Science, Sogang University, Seoul, South Korea

SUPPLEMENTARY INFORMATION

Table S1. Species composition of the phylum Bacteroidetes in the fecal samples of *V. vulnificus*-infected mice

Table S2. Bacterial strains and plasmids used in this study

Table S3. Oligonucleotides used in this study

Figure S1. Survival of *B. vulgatus* in the presence of various kinds of bacterial cells

Figure S2. Release of DiSC3(5) from *B. vulgatus* cells

Figure S3. Effect of cFP on the abundance of *B. vulgatus*, *P. goldsteinii*, and *L. reuteri* in mouse fecal samples

Figure S4. Alignment of the amino acid sequences of diverse ObgEs

Table S1. Species composition of the phylum Bacteroidetes in the fecal samples of *V. vulnificus*-infected mice

Name [a]	Experiment 1		Experiment 2	
	**Control mice** Avg (sd)	**Dead mice** Avg (sd)	**Control mice** Avg (sd)	**Dead mice** Avg (sd)
Bacteroides vulgatus	52.50 (9.450)	33.15 (6.502)	36.34 (1.523)	27.76 (1.741)
Parabacteroides goldsteinii	1.812 (0.962)	0.674 (0.183)	0.905 (0.405)	0.354 (0.003)
Bacteroides caccae	4.299 (2.484)	1.222 (0.031)	1.764 (1.477)	1.566 (1.639)
*Bacteroides*_uc	0.056 (0.030)	0.053 (0.023)	0.025 (0.035)	nd [b]
*Parabacteroides*_uc	0.018 (0.025)	nd	nd	nd
HQ821223_s	0.216 (0.132)	0.067 (0.047)	nd	nd
DQ799357_s	0.016 (0.022)	0.009 (0.015)	nd	nd
AB626927_s	2.887 (4.084)	0.571 (0.963)	4.306 (2.545)	nd
AB626927_g_uc	0.246 (0.348)	0.047 (0.075)	0.295 (0.347)	nd
Muribaculaceae_uc	0.035 (0.050)	nd	0.05 (0.07)	nd

[a] The annotation of species was followed by EzBioCloud (1).

[b] The species occupied more than 0.01% of the total microbiota in a mouse fecal sample were listed in this table.

Reference

1. Yoon SH, Ha SM, Kwon S, et al. Introducing EzBioCloud: A taxonomically united database of 16S rRNA gene sequences and whole-genome assemblies. Int J Syst Evol Microbiol. 2017;67:1613-1617. doi:10.1099/ijsem.0.001755

Table S2. Bacterial strains and plasmids used in this study

Strain/plasmid	genotype	source/reference
V. vulnificus		
MO6-24/O	Clinical isolate	1
ΔicmF	MO6-24/O, deletion mutant of T6SS structural component coding gene, *icmF*	This study
ΔllcA	MO6-24/O, deletion mutant of low level cFP producer A coding gene, *llcA*	2
B. vulgatus		
ATCC8482	Human fecal isolate	ATCC
DSM28735	TNFdeltaARE/+ C57BL/6 mouse caecal isolate	DSMZ
MGM001	Fecal isolate	This study
**Mouse Gut Microbes**		
P. goldsteinii MGM002	Fecal isolate	This study
L. johnsonii MGM003	Fecal isolate	This study
L. johnsonii MGM004	Fecal isolate	This study
L. reuteri MGM005	Fecal isolate	This study
L. reuteri MGM006	Fecal isolate	This study
L. murinus MGM007	Fecal isolate	This study
L. murinus MGM008	Fecal isolate	This study
L. intestinalis MGM009	Fecal isolate	This study
E. coli		
DH5α	(ϕ80 *lacZ ΔM15*) *recA1 endA1 gyrA96 relA1 thi-1 hsdR17*(r_K^- m_K^-) *supE44 deoR* Δ(*lacZYA-argF*) *U169*	Lab collection
S17-1 λ*pir*	*Tpr Smr recA thi pro hsdR*$^-$*M,*$^+$*RP4:2-Tc:Mu:Km Tn7, λpir*	3
BL21(DE3)	*E. coli* strain B F$^-$ *dcm ompT hsdS* (rB$^-$ mB$^-$) *galλ* (DE3)	Invitrogen
V. cholerae		

ATCC14033	Serovar O1, biotype ElTor, serotype Inaba	ATCC
V. parahaemolyticus		
RIMD2210633	Serotype O3:K6	ATCC/BAA-238
**Plasmid**		
pBlueScript II SK(+)	Cloning vector; Ap^{R}, *lac* promoter (*lacZ*), f1, ColE1	Stratagene
pDM4	Suicide vector, OriR6K, Cm^{R}	4
pSK-icmF up	pBluescript II SK(+) with 565-bp *V. vulnificus icmF*	This study
	upstream region	This study
pSK-icmF updown	pSKrsfup with 749-bp V. vulnificus *icmF*	This study
	downstream region	
pDM4-ΔicmF	pDM4, 1,314-bp SalI/XbaI fragment of	This study
	icmF updown, Cm^{R}	
pET-28a	Expression vector for a histidine-tagged protein, Km^{R}	Novagen
pET28a-obgE	pET-28a, 1185-bp *obgE* coding region, Km^{R}	This study
pBAD/*Myc*-His B	Cloning vector to make C-terminal Myc 6x His-tagged	Invitrogen
	proteins expressed under arabinose control, Ap^{R}	
pBAD-*obgE*$_{Bv}$	pBAD/Myc-His, 1185-bp NcoI/KpnI fragments	This study
	of *obgE* of *B. vulgatus*	

References

1. Wright AC, Simpson LM, Oliver JD, Morris JG Jr. Phenotypic evaluation of acapsular transposon mutants of *Vibrio vulnificus*. Infect Immun. 1990;58:1769-1773. doi: 10.1128/IAI.58.6.1769-1773.1990.

2. Kim K, Kim NJ, Kim SY, Kim IH, Kim KS, Lee GR. Cyclo(Phe-Pro) produced by the human pathogen *Vibrio vulnificus* inhibits host innate immune responses through the NF-κB pathway. Infect Immun. 2015;83:1150-1161. doi:10.1128/IAI.02878-14

3. Simon R, Priefer U & Pühler A. A broad host range mobilization system for *in vivo* genetic engineering: Transposon mutagenesis in Gram negative bacteria. Nat Biotechnol 1983;1:784–791 doi:10.1038/nbt1183-

4. Milton DL, O'Toole R, Horstedt P, Wolf-Watz H. Flagellin A is essential for the virulence of *Vibrio anguillarum*. J Bacteriol. 1996;178:1310-1319. doi:10.1128/jb.178.5.1310-1319.1996

Table S3. Oligonucleotides used in this study

Name	Sequence (5′ to 3′) of oligonucleotide [a]
**Primers for qPCR of mouse fecal microbiota**	
Bv-F	GCATCATGAGTCCGCATGTTC
Bv-R	TCCATACCCGACTTTATTCCTT
785-F	GGATTAGATACCCTGGTA
907-R	CCGTCAATTCMTTTRAGTTT
Lr-F	GATTGACGATGGATCACCAGT
Lr-R	CATCCCAGAGTGATAGCCAA
Pg_49-69F	GCAGCACGATGTAGCAATACA
Pg_192-169R	TTAACAAATATTTCCATGTGGAAC
**Primers for construction of *ΔicmF* mutant**	
icmFupF Sal	GCGTCGACGATCAGACCTTTAGTCAGTTAATG
icmFupRPst	AACTGCAGCCCCAATAAACGTTTAATCTTTGCC
icmFdownFPst	AACTGCAGCCATTTTGGTCGAGTGATATGGAG
icmFdownRXba	GCTCTAGACTCCAACTCATTCGATCACCG
**Primers for qRT-PCR**	
obgE_RT-F	GATTATGTGAAGATATATTGC
obgE_RT-R	CTTCAGGTGAAGCAATGTCC
recA_RT-F	CCATTCGCAGCAAGTCCGAG
recA_RT-R	CCCCTAATGCGGCGTTCAAGG
cidA_RT-F	GCGCCATTTTATTTGGCTG
cidA_RT-R	GCCCAGATTAGCAACCAAG
gap_RT-F	CGTATTGGGTTACCTGAAG
gap_RT-R	ACCGATTTCGTTGTCATACC
**Primers for overexpression of recombinant proteins**	
ObgE_over-F	CGGGATCCATGGCTGAATCGAATTTTG

ObgE_over-R	ACGCGTCGACTCATTTCTTTTCCTCTTC
RecA_over-F	CGGGATCCATGGCAAAAAAAGATAAC
RecA_over-R	ACGCGTCGACTTATTCTTGTTCTTTCAG
**Primers for arabinose dependent expression of ObgE**	
ObgE_pBAD-F	CATGCCATGGATGGCTGAATCGAATTTTG
ObgE_pBAD-R	GGTCTAGATCATTTCTTTTCCTCTTC

[a] Restriction sites are underlined and their usages in cloning experiments are described in "Methods".

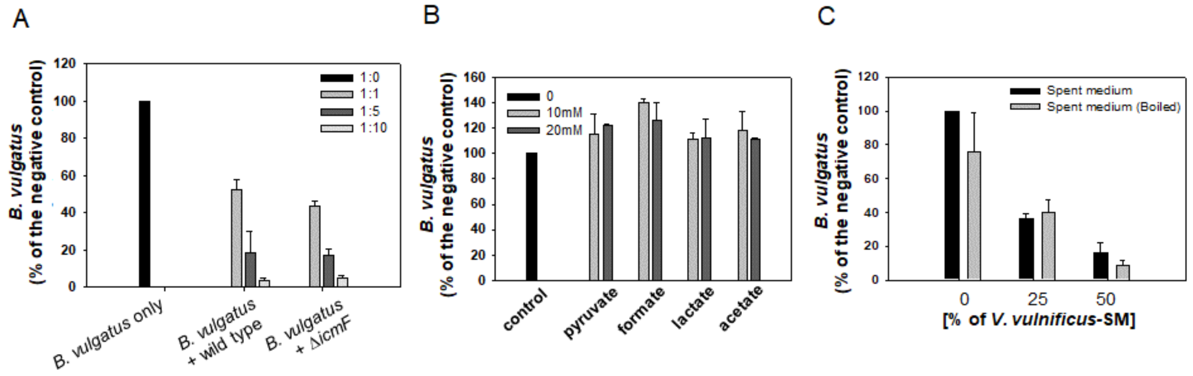

Figure S1. Survival of *B. vulgatus* in the presence of various kinds of bacterial cells

(A) Survival of *B. vulgatus* in the presence of a T6SS-deficient *V. vulnificus*. To examine the possibility of direct cell-to-cell interaction in *B. vulgatus* death via T6SS of *V. vulnificus*, a mutant *V. vulnificus* deficient in one of the main T6SS components (Δ*icmF*), was mixed with *B. vulgatus* at ratios (*B. vulgatus*:*V. vulnificus*) of 1:1, 1:5, and 1:10. *B. vulgatus* CFUs were enumerated as described in Fig. 2C.

[Method] **Construction of an *icmF* gene-deleted *V. vulnificus***: To obtain the *ΔicmF V. vulnificus*, a 565 bp DNA fragment containing the upstream region of *icmF* was amplified using the primers icmFupF and icmFupR (Table S3). The PCR product was cloned into pBluescript SKII(+) to produce pSK*icmF*up. A 749 bp DNA fragment containing the downstream region of *icmF*, which was amplified using primers icmFdownF and icmFdownR, was cloned into the corresponding sites of pSK*icmF*up to produce pSK*icmF*up/down. A 1,314 bp DNA fragment of pSK*icmF*up/down digested with SalI and XbaI was ligated into pDM4 to generate pDM4-Δ*icmF*. *E. coli* SM10λpir carrying pDM4-Δ*icmF* was conjugated with *V. vulnificus* MO6-24/O and the exconjugants were then selected on thiosulfate citrate bile sucrose (TCBS) agar plates supplemented with 2 μg/ml chloramphenicol. While subculturing an exconjugant *V. vulnificus* carrying pDM4-Δ*icmF* in the absence of an antibiotic, the colonies with characteristics indicating a double homologous recombination event (resistance to 5% (w/v) sucrose and sensitivity to 2 μg/ml chloramphenicol) were isolated, as previously described (1). The deletion of *icmF* in its genome was further confirmed by PCR using a primer set of icmFupF and icmFdownR. Information regarding the primers used in this study is listed in Table S3.

(B) Effect of metabolites on the survival of *B. vulgatus*. To examine the possible role of main metabolites produced by *V. vulnificus*, four major fermentation products were added to *B. vulgatus* suspension in M9 medium. After 6 h of anaerobic incubation at 37°C, *B. vulgatus* CFUs were enumerated as described in Fig. 2C.

(C) Survival of *B. vulgatus* in the presence of heat-treated SM of *V. vulnificus*. To examine the possible role of the protein(s) in the *V. vulnificus*-SM in the death of *B. vulgatus*, the SM was heated at 95°C for 5 min. Two different concentrations (0.25x and 0.5x-dilutions in the fresh LBS) of heat-treated SM were added to the *B. vulgatus* suspension in LBS medium. After 6 h of anaerobic incubation at 37°C, *B. vulgatus* CFUs were enumerated as described in Fig. 2C.

Reference

1. Park KJ, Kang MJ, Kim SH, et al. Isolation and characterization of *rpoS* from a pathogenic bacterium, *Vibrio vulnificus*: Role of σ^S in survival of exponential-phase cells under oxidative stress. J Bacteriol. 2004;186:3304-3312. doi:10.1128/JB.186.11.3304-3312.2004

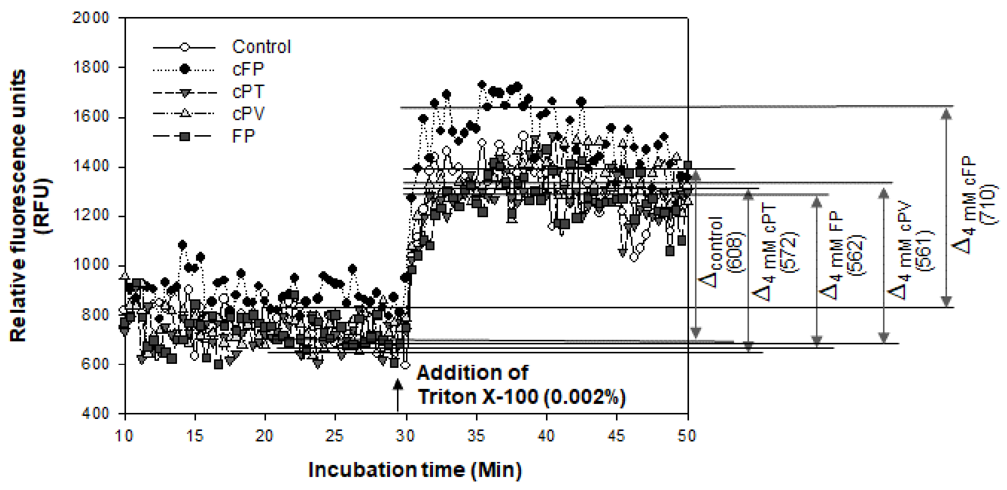

Figure S2. Release of DiSC3(5) from *B. vulgatus* cells

Various kinds of dipeptides: As described in Figure 7, DiSC3(5) was incorporated into membranes of *B. vulgatus* cells, which had been exposed to 4 mM FP, cPT, and cPV. After unincorporated dyes were washed out, the relative fluorescence units (RFUs) from DiSC3(5) associated with cells were measured using a fluorometer at an excitation wavelength of 622 nm and emission wavelength of 670 nm. At 30 min (as indicated with a black vertical arrow) cells were treated with 0.002% Triton X-100. The released fluorescence from the Triton X-100-treated cells (termed by $\Delta_{0.002\%}$) was obtained by subtracting the values of basal RFU (averaged RFUs for 10 min before treatment of 0.002% Triton X-100) from the maximal RFU (averaged RFUs for 10 min after treatment of 0.002% Triton X-100). As controls, the cells treated with 0 ($\Delta_{control}$) and 4 mM cFP ($\Delta_{4mM\ cFP}$) were included in the assays.

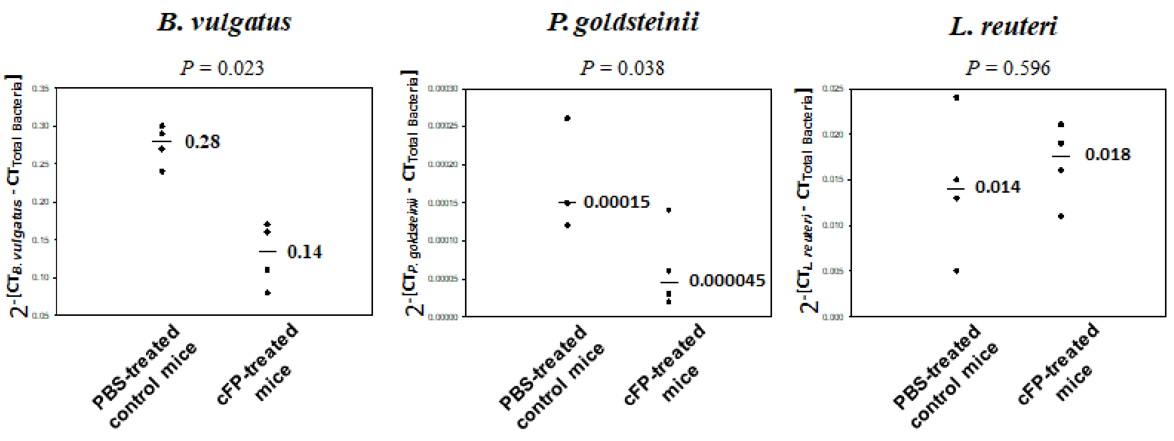

Figure S3. Effect of cFP on the abundance of *B. vulgatus*, *P. goldsteinii*, and *L. reuteri* in mouse fecal samples

Four fecal samples, which have been used for estimating the *B. vulgatus* abundance shown in the Figure 9A, were selected and further analyzed to estimate the abundance of other bacterial species belonged to Bacteroidetes and Firmicutes. The total DNA extracts were subjected to q-PCR using a primer set specific to 16S rDNA sequences of *P. goldsteinii* (Pg_49-69F and Pg_192-169R, [1]) or *L. reuteri* (Lr-F and Lr-R, [2]) (see the Additional file 8). The abundance of each 16S rDNA was normalized with the abundance of the total bacterial 16S rDNAs, which were amplified using the universal primer set for eubacterial 16S rDNA (785-F and 907-R, [3]). The relative abundance was presented by the values of $2^{-[CT\ \textit{P. goldsteinii}\ -\ CT\ \mathrm{Total\ Bacteria}]}$ or $2^{-[CT\ \textit{L .reuteri}\ -\ CT\ \mathrm{Total\ Bacteria}]}$ with their median numbers. Statistical analysis was performed using the Student's *t*-test and the resulting *P*-values were provided.

References

1. Gomes-Neto JC, Mantz S, Held K et al. A real-time PCR assay for accurate quantification of the individual members of the Altered Schaedler Flora microbiota in gnotobiotic mice. J Microbiol Methods. 2017;135:52-62. doi: 10.1016/j.mimet.2017.02.003

2. Kim E, Yang SM, Lim B et al. Design of PCR assays to specifically detect and identify 37 *Lactobacillus* species in a single 96 well plate. BMC Microbiol. 2020;20:96. doi: 10.1186/s12866-020-01781-z.

3. Ziesemer KA, Mann AE, Sankaranarayanan K, et al. Intrinsic challenges in ancient microbiome reconstruction using 16S rRNA gene amplification. Sci Rep. 2015;5:16498. doi:10.1038/srep16498

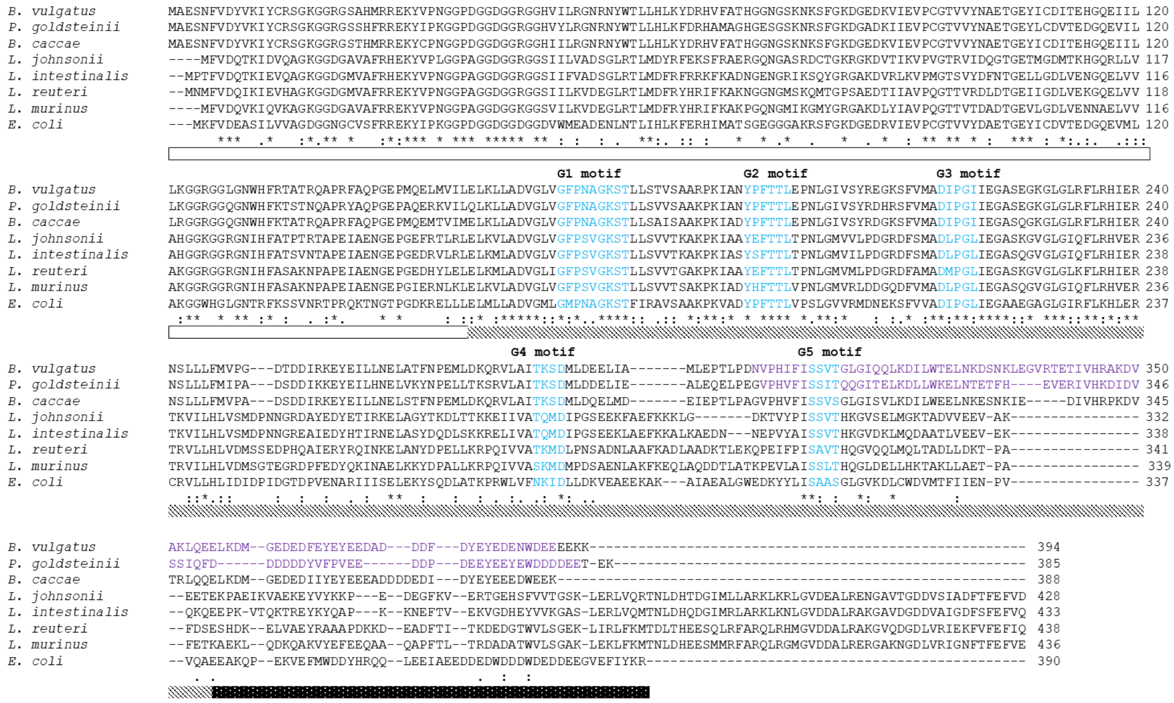

Figure S4. Alignment of the amino acid sequences of diverse ObgEs

Amino acid sequences of ObgE derived from several bacterial species were aligned using Clustal Omega (https://www.ebi.ac.uk/Tools/msa/clustalo/). The accession numbers of the corresponding genes in GenBank are as follows: *B. vulgatus*, WP005839602; *P. goldsteinii*, WP129735263; *B. caccae*, WP_005679369; *L. johnsonii*, WP061399959; *L. intestinalis*, WP057811265; *L. reuteri*, WP153700693; *L. murinus*, WP148458313; and *E. coli*, QGJ14529. Three domains were proposed in the *E. coli* ObgE, which consists of Obg (amino acid residues from 1 to 157; designated with an open bar), GTP-binding (amino acid residues from 158 to 340; designated with a slashed bar), and C-terminal domains (designated with a dotted bar) (1). The Obg domain, rich in glycine residues, is a highly conserved N-terminal domain that plays a role in scaffolding. The GTP-binding domain contains five G motifs (designated by G1 to G5) and two switch elements for the binding of GTP/GDP and hydrolysis of GTP. However, the C-terminal domain is poorly conserved. The G1-G5 motives found in all ObgE are indicated by blue-colored letters and the putative DNA Pol Phi domain found in ObgE of *B. vulgatus* and *P. goldsteinii* are indicated by purple-colored letters. Identical, conserved, and semiconserved amino acid residues are denoted by an asterisk (*), colon (:), and period (.), respectively.

Reference

1. Gkekas S, Singh RK, Shkumatov AV et al. Structural and biochemical analysis of *Escherichia coli* ObgE, a central regulator of bacterial persistence. J Biol Chem. 2017;2925871-5883. doi:10.1074/jbc.M116.761809
